# Supplementary material for: Precision Functional Mapping of Imagined and Experienced Pain
Source: bioRxiv. 2026 Sep 1:2025.08.06.668906. Preprint. [Version 2] doi: 10.1101/2025.08.06.668906 (PMC13684548; doi:10.1101/2025.08.06.668906)
Supplement: Supplementary file 1 [file media-1.pdf]

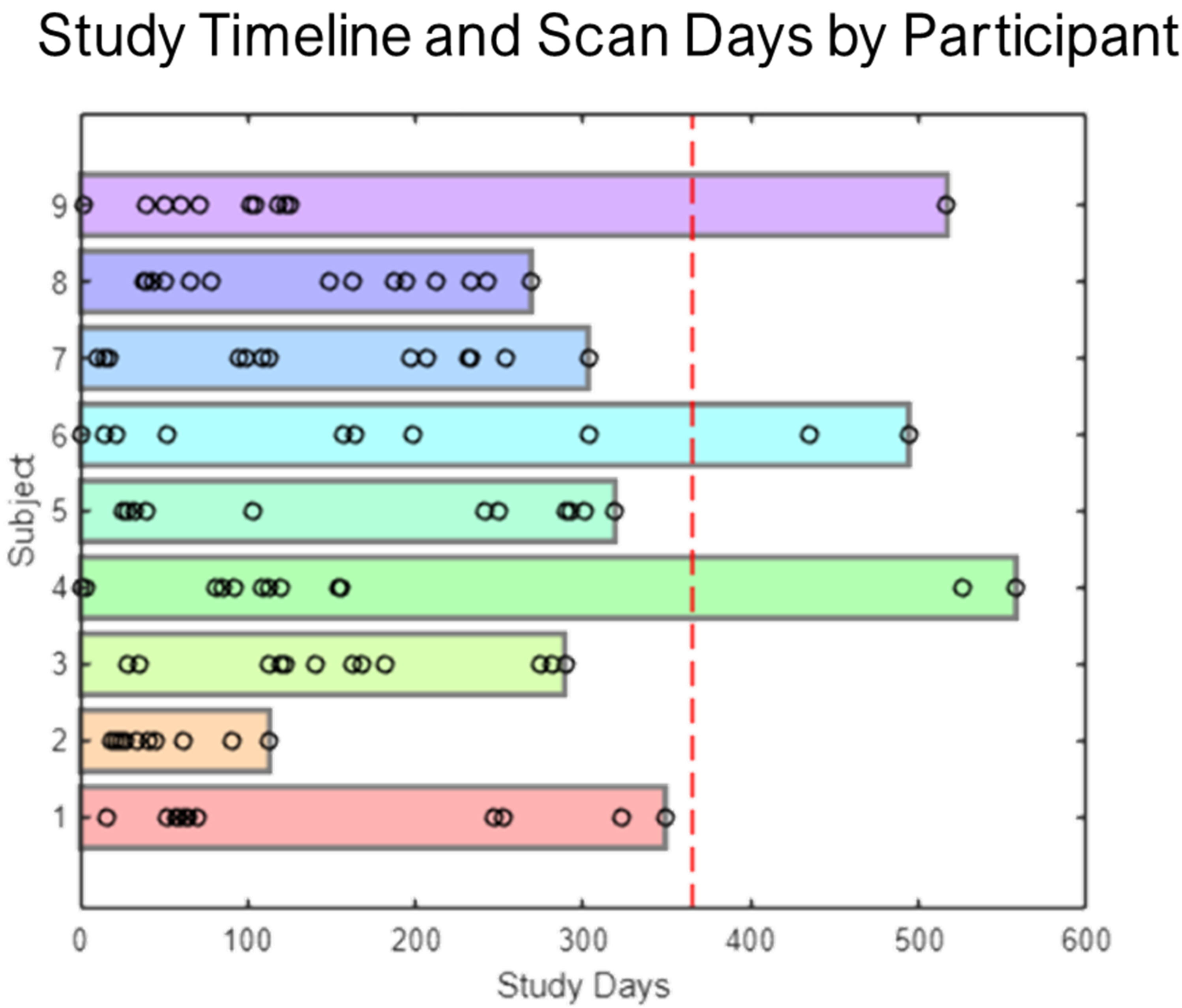

Supplemental Figure 1: Study timeline and scan days by participant. Horizontal bars indicate the total calendar span (in days) over which each participant was involved in the study. Circles denote individual fMRI scanning days. Participants were scheduled for 10 scanning sessions; however, some participants completed additional sessions due to re-scanning, whereas others completed multiple sessions on the same day, resulting in different numbers of distinct scan days. The dashed red line indicates one year (365 days) as a point of reference.

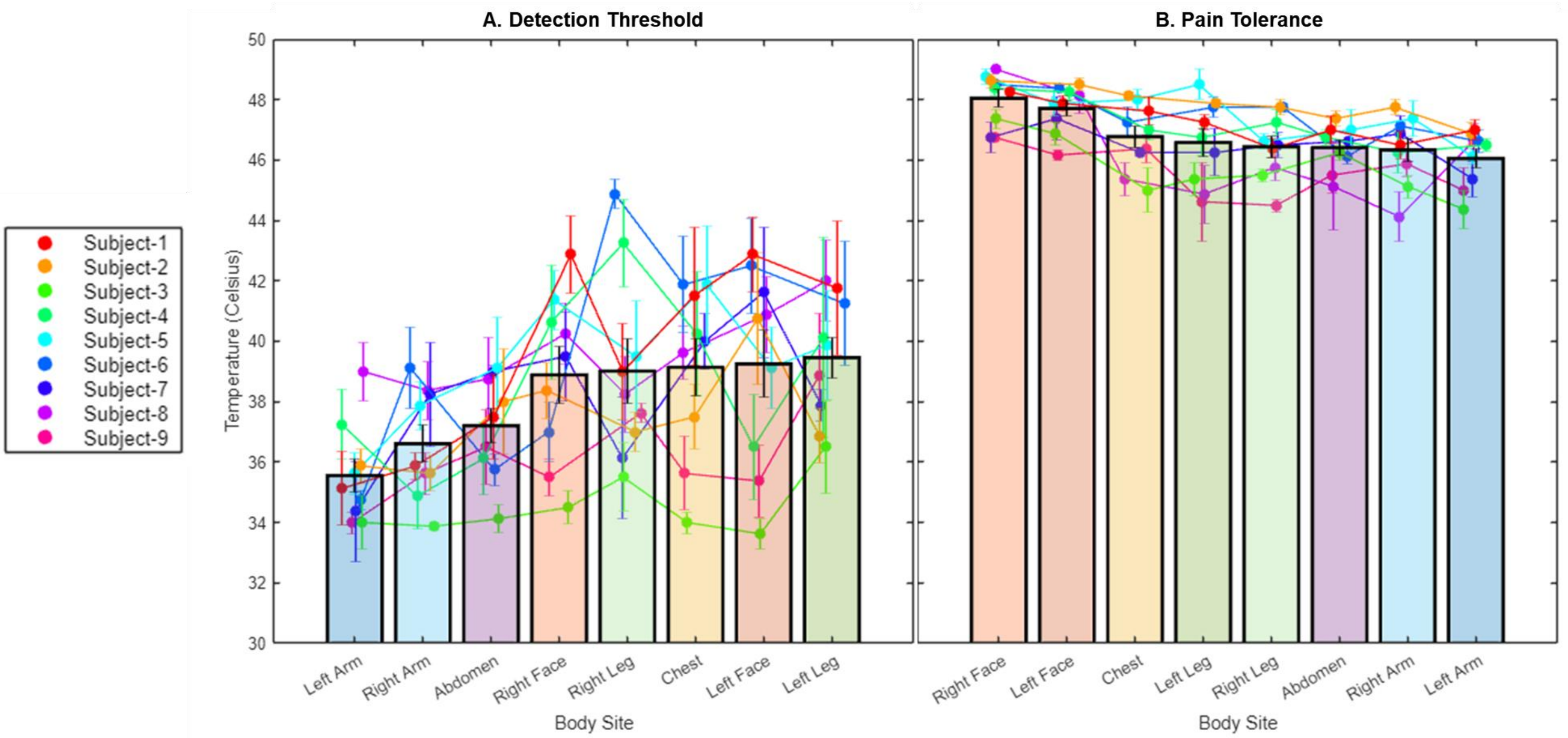

Supplemental Figure 2: Participant-level thermal detection thresholds and pain tolerance across body sites. Participant-level (A) thermal detection thresholds and (B) pain tolerance at each body site. Bars show group mean ± SEM across n = 9 participants; dots show individual participants.

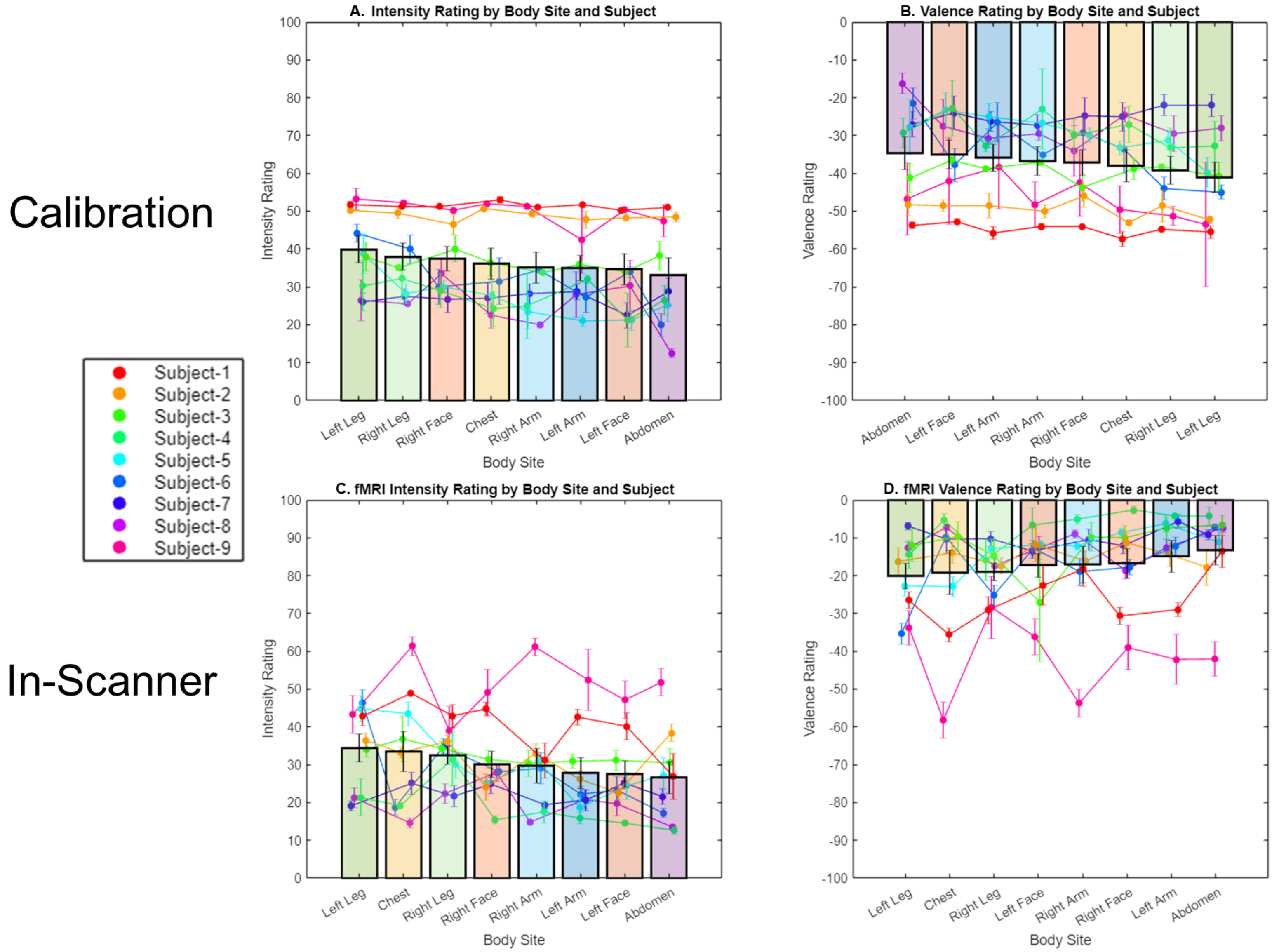

Supplemental Figure 3: Participant-level intensity and valence ratings during calibration and scanning. Participant-level ratings during calibration for (A) intensity and (B) valence, and during fMRI scan-sessions for (C) intensity and (D) valence ratings. Bars show group mean ± SEM across n = 9 participants; dots show individual participants.

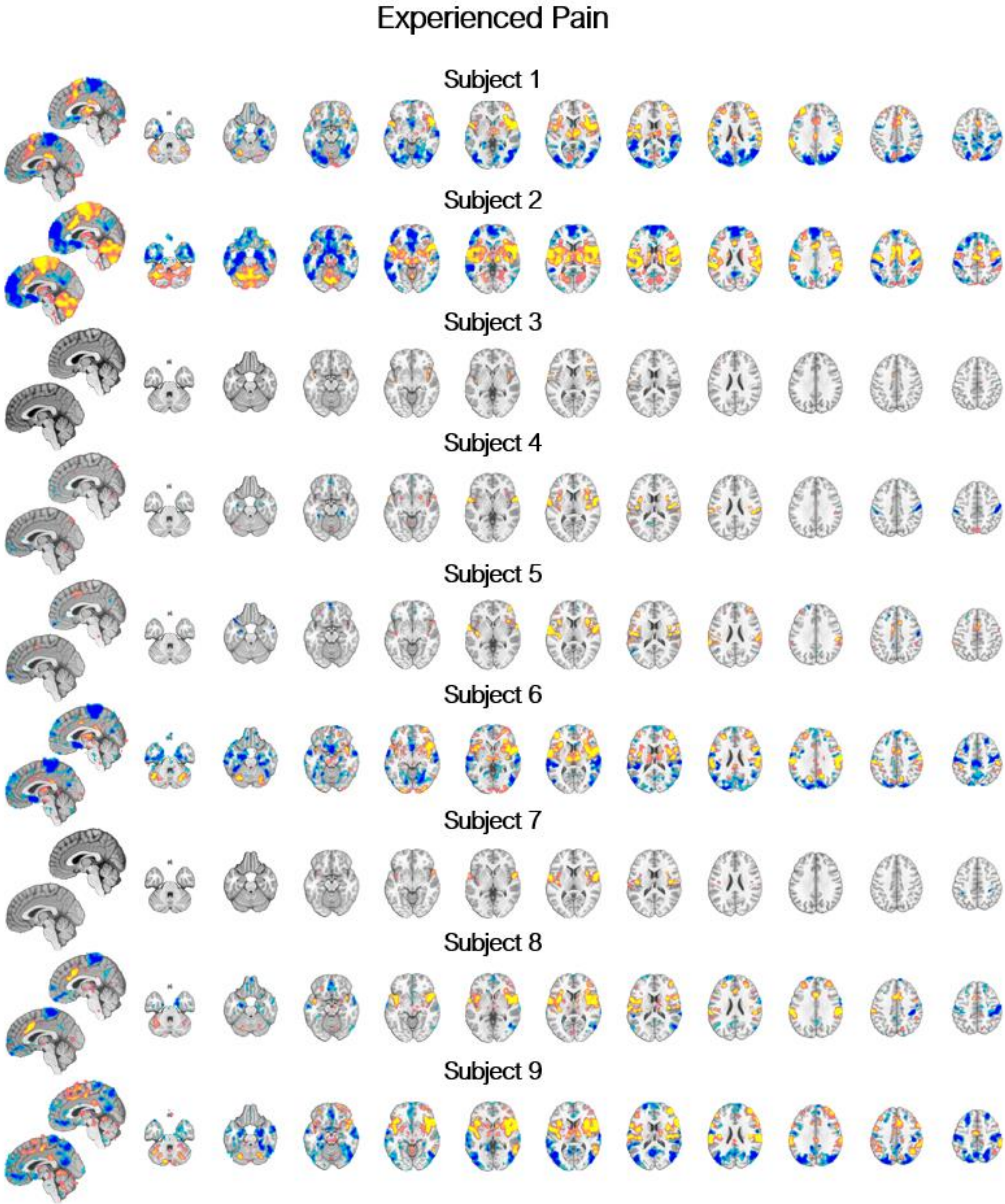

Supplemental Figure 4: Participant-level experienced pain contrast (hot–warm). Participant-level experienced pain contrast (hot-warm). FDR q < .05.

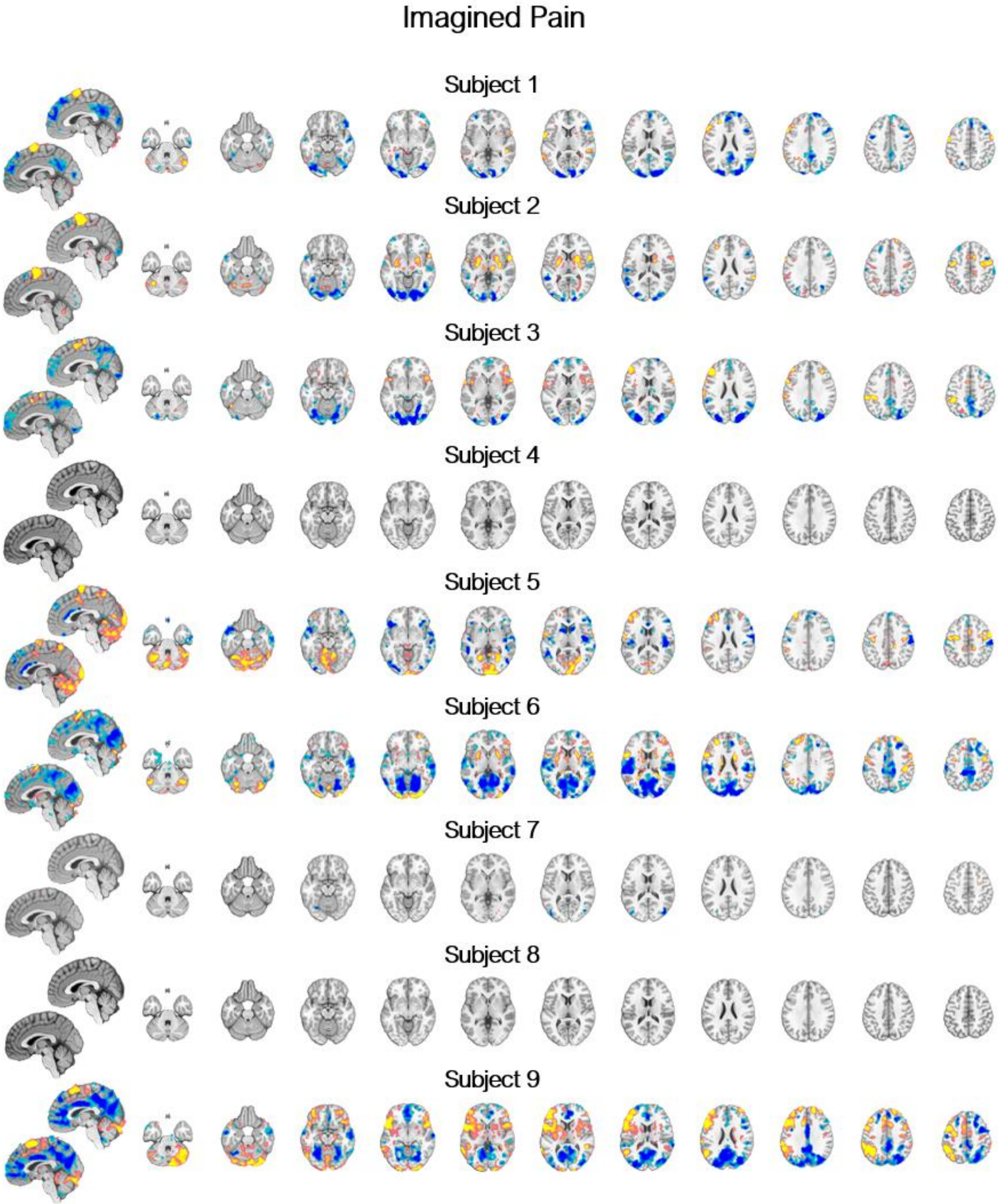

Supplemental Figure 5: Participant-level imagined pain contrast (imagine–warm). Participant-level imagined pain contrast (imagine-warm). FDR q < .05.

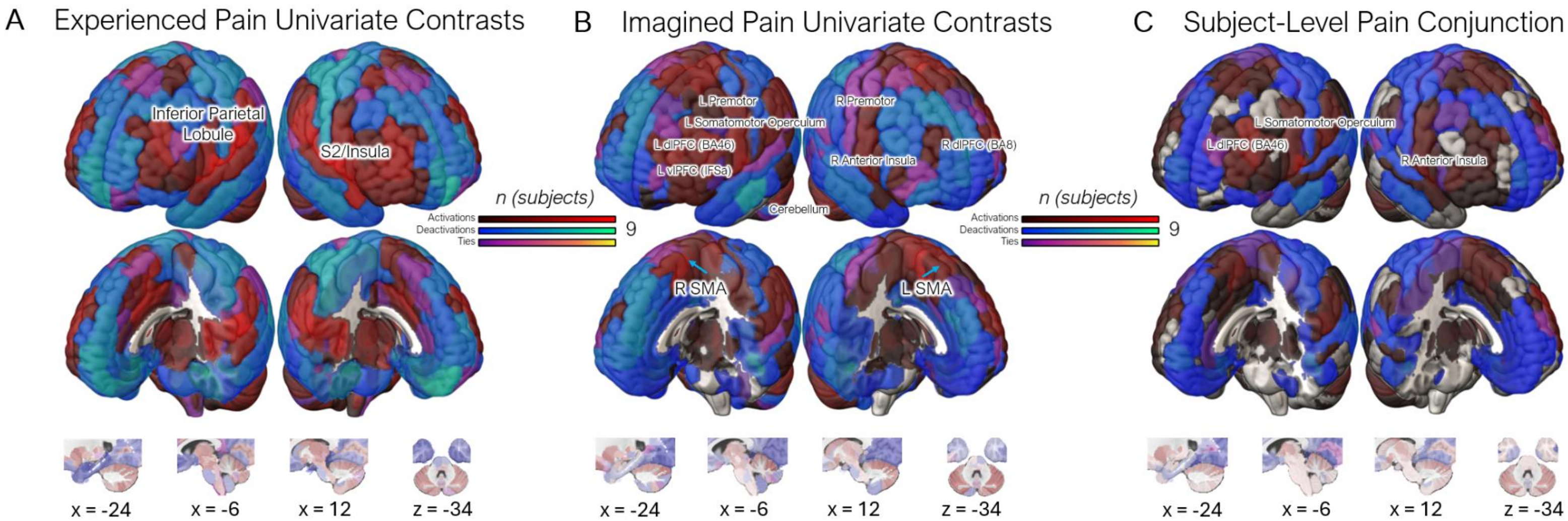

Supplemental Figure 6: Count maps of FDR-thresholded participant-level univariate activations of regions. Each region required a coverage threshold of 5% of total region voxels activated or deactivated by the participant to be counted toward the respective counts of "activated" or "deactivated" regions FDR q < .05. Regions are colored in accordance to whether they are activated or deactivated by the majority of participants, or if there are an equal number of participants that are activating and deactivating.

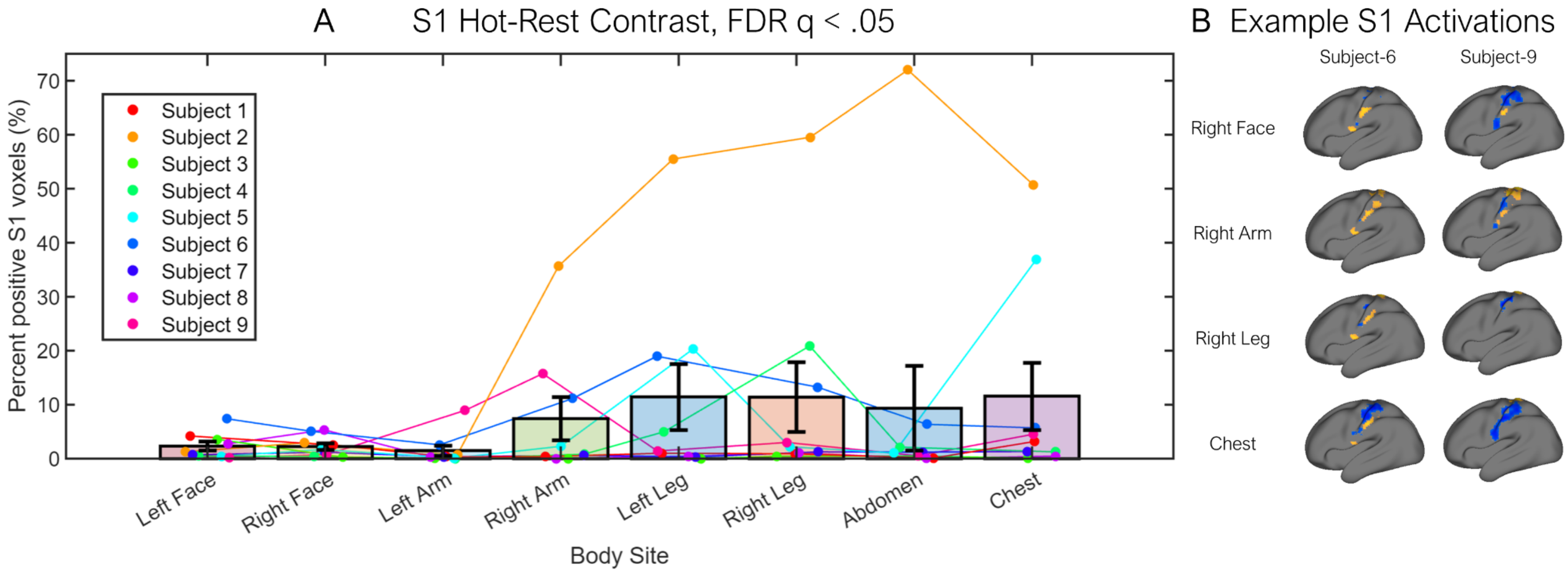

Supplemental Figure 7: Body-site–specific S1 activation and inter-individual variability. (A) Percentage of S1 voxels showing significant activation for the Hot–Rest contrast (FDR $q < 0.05$) is plotted for each body site. Bars indicate the group mean ± SEM across subjects, with individual subjects shown as colored points connected across body sites (colors denote subjects). S1 activation was observed in all subjects for the left face, right face, right leg, abdomen, and chest, whereas activation was observed in 8/9 subjects for the left arm and left leg, and in 7/9 subjects for the right arm. (B) Subject-level surface maps for example subjects 6 and 9 illustrating focal, body-site–specific S1 activation for the Hot–Rest contrast (FDR $q < 0.05$), with substantial inter-individual variability in the spatial extent of activation. Bars show group mean ± SEM across n = 9 participants.

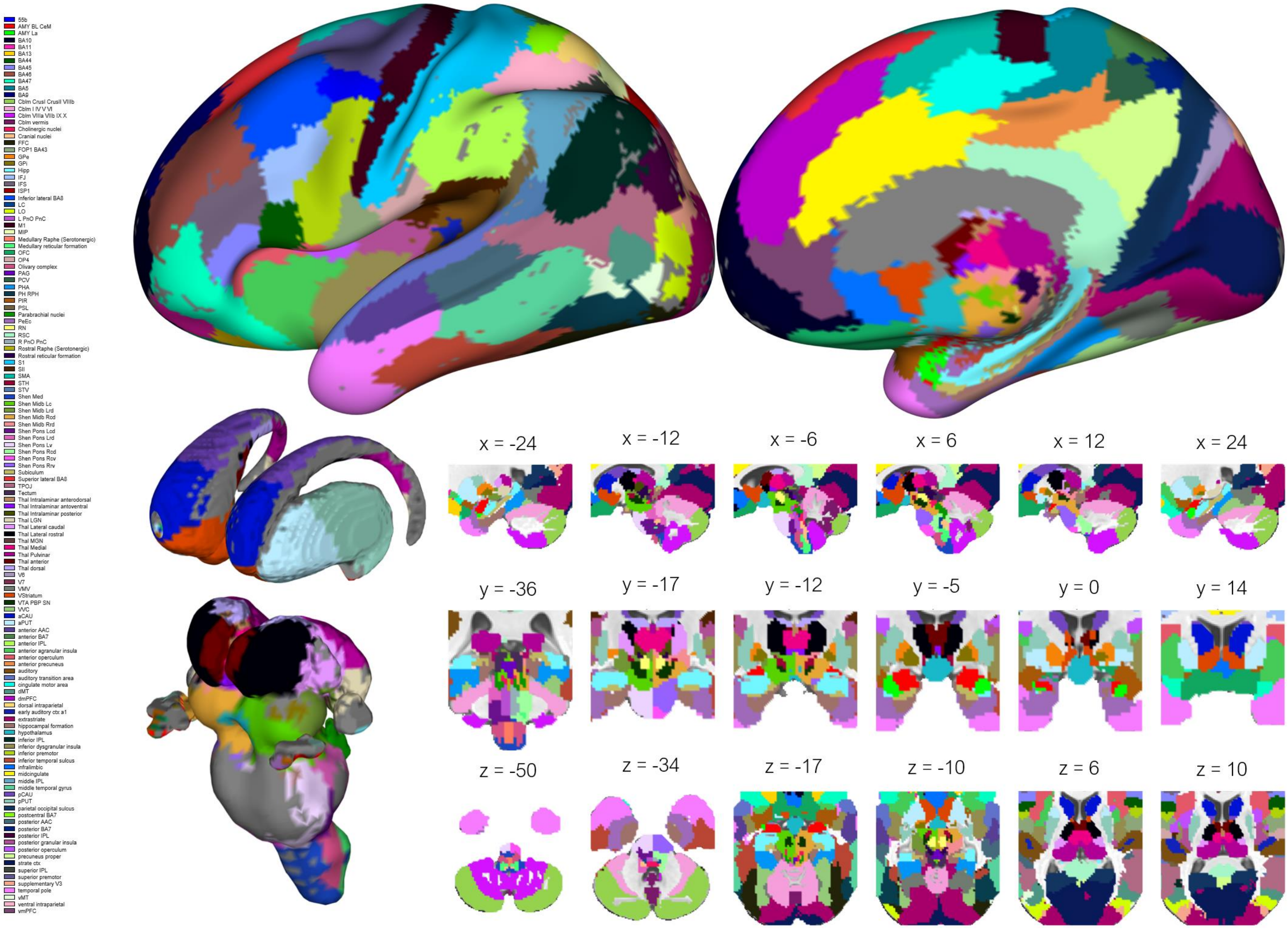

Supplemental Figure 8: The 131 Region CANLab 2024 Atlas.

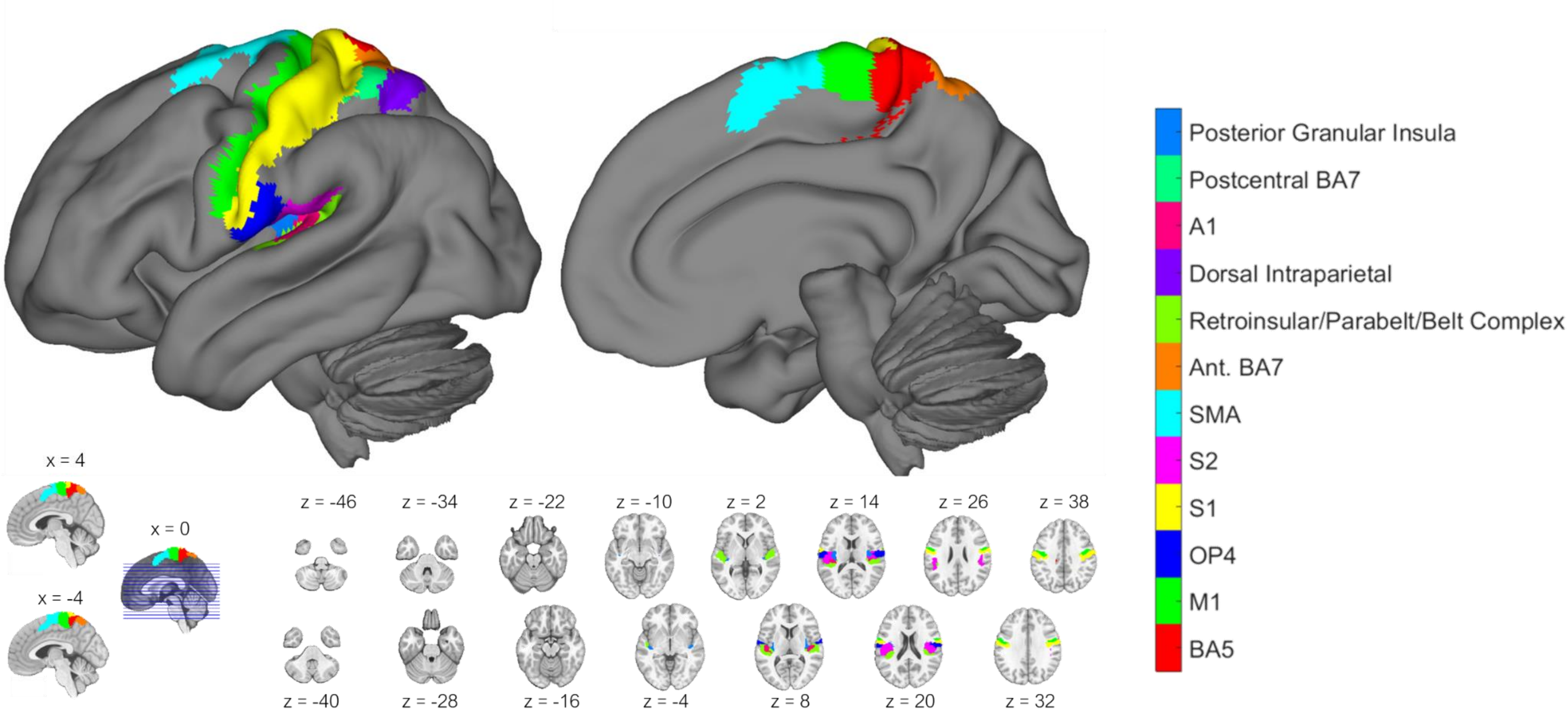

Supplemental Figure 9: FDR-corrected nociceptive body site selective parcels from the CANLab 2024 Atlas. BA: Brodmann Area. VTA: Ventral Tegmental Area. LGN: Lateral Geniculate Nucleus. IL: Intralaminar. SMA: Supplementary Motor Area. S2: Secondary Somatosensory Cortex. S1: Primary Somatosensory Cortex. OP4: Opercular Area 4. M1: Primary Motor Cortex.

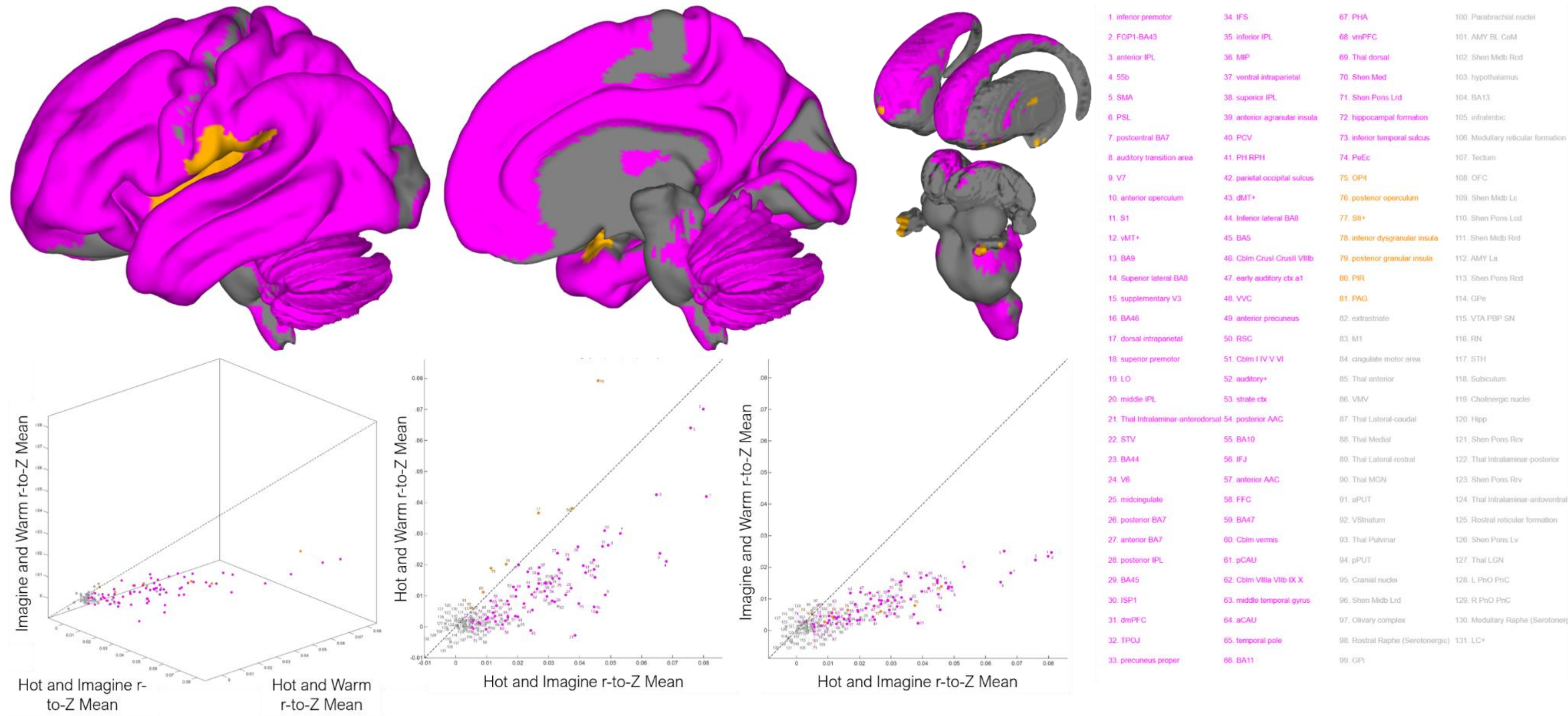

Supplemental Figure 10: Regional dominance of cross-condition representational similarity. Regions were colored by their highest significant condition-pair similarity. A region is colored by that dominant pair only if its dominant similarity was itself significantly greater than zero across participants (one-sample t-test, two-tailed P < .05); otherwise it is shown in gray. In most parcels (74/131), the dominant, significant similarity was between the Hot and Imagine conditions (magenta), indicating that imagined-pain activity patterns most closely resembled those evoked by experienced pain. Seven parcels (amber) were instead most similar between Hot and Warm. These included the periaqueductal gray (PAG), piriform cortex (PIR), and a cluster of opercular–insular somatosensory regions (SII+, OP4, posterior operculum, inferior dysgranular insula, and posterior granular insula). This is consistent with experienced thermal input dominating these early somatosensory areas. No region showed a significant dominant similarity between Imagine and Warm. In the scatter plots, each point is one region colored by its dominant significant pair, and the dashed diagonal marks the identity line, along which the two cross-condition similarities on the axes (or, in the three-dimensional plot, all three) are equal. Gray regions are those whose highest cross-condition similarity did not reach statistical significance.

Supplemental Table S1. Calibration temperatures and rating metrics

Body Site	Detection Threshold		Pain Tolerance		Calibration Valence		Calibration Intensity		Scanner Valence		Scanner Intensity	
	M	SE	M	SE	M	SE	M	SE	M	SE	M	SE
Left Face	39.25	1.11	47.71	0.26	-35.00	4.71	34.68	2.62	-17.19	3.14	27.57	3.43
Right Face	38.89	0.93	48.04	0.29	-37.14	4.20	37.47	2.77	-16.72	3.84	30.10	3.52
Left Arm	35.56	0.55	46.06	0.31	-35.83	3.56	35.00	3.01	-14.85	4.22	27.83	4.08
Right Arm	36.61	0.61	46.33	0.38	-36.75	3.21	35.17	2.16	-17.04	4.83	29.72	4.54
Left Leg	39.46	0.69	46.58	0.46	-41.06	4.61	39.89	3.31	-20.07	3.35	34.39	3.70
Right Leg	39.01	1.05	46.44	0.36	-39.19	2.93	37.94	1.99	-19.01	2.29	32.51	2.35
Chest	39.14	0.94	46.78	0.37	-37.97	3.38	36.14	3.04	-19.19	5.81	33.49	5.24
Abdomen	37.21	0.57	46.42	0.25	-34.64	4.27	33.14	2.87	-13.27	3.84	26.64	4.20

Note. All means are averages across N = 9 participants.

Supplementary Table S2. Univariate analysis through voxelwise robust regression.

Structure	Region	Parcel	Network	Volume	MNI Coordinates X	Y	Z	Experienced Pain (Hot-Warm) T	Activated (n)	Deactivated (n)	Imagined Pain (Imagine-Warm) T	Activated (n)	Deactivated (n)	Pain Conjunction Activated (n)	Deactivated (n)
Amygdala L	MTL AMY BL L	AMY BL CeM L	subctx	880	-21	-4	-20	-3	0	6	9.01	1	0	0	0
Amygdala L	MTL AMY CeM L	AMY BL CeM L	subctx	520	-20	-5	-16	5.02	1	4	5.62	2	0	0	0
Amygdala L	MTL AMY La L	AMY La L	subctx	736	-28	-3	-23	-3.57	0	6	-3.01	0	1	0	1
Amygdala R	MTL AMY BL R	AMY BL CeM R	subctx	816	22	-4	-19	-3.91	0	5	10.31	0	0	0	0
Amygdala R	MTL AMY CeM R	AMY BL CeM R	subctx	608	20	-5	-16	4.1	1	3	3.21	2	0	0	0
Amygdala R	MTL AMY La R	AMY La R	subctx	784	28	-2	-24	-5.03	0	5	-1.96	0	0	0	0
CAU L	BG CAU DA L	aCAU L	subctx	1832	-14	19	6	-4.05	0	2	-3.34	1	1	0	0
CAU L	BG CAU VA L	aCAU L	subctx	1144	-10	12	5	6.72	1	1	3.21	1	1	1	0
CAU L	BG CAU body L	pCAU L	subctx	1168	-13	6	17	8.06	2	0	1.92	2	1	0	0
CAU L	BG CAU tail L	pCAU L	subctx	1408	-16	-9	23	6.98	2	0	5.86	1	0	1	0
CAU R	BG CAU DA R	aCAU R	subctx	2184	14	20	7	6.32	1	2	-7.66	2	1	0	0
CAU R	BG CAU VA R	aCAU R	subctx	1008	10	13	4	4.65	4	1	-5.02	1	1	1	0
CAU R	BG CAU body R	pCAU R	subctx	1248	13	7	17	10.04	4	0	-5.47	3	1	1	0
CAU R	BG CAU tail R	pCAU R	subctx	1336	16	-9	23	4.74	4	0	3.29	3	1	2	0
Cblm cortex L	Cblm CrusI L	Cblm CrusI CrusII VIIIb L	subctx	15848	-36	-68	-32	8.54	5	0	-6.83	5	1	3	0
Cblm cortex L	Cblm CrusII L	Cblm CrusI CrusII VIIIb L	subctx	11888	-27	-74	-42	-7.5	4	0	-5	1	3	0	0
Cblm cortex L	Cblm I IV L	Cblm I IV V VI L	subctx	3480	-8	-45	-16	-6.94	2	0	3.15	3	0	2	0
Cblm cortex L	Cblm IX L	Cblm VIIIa VIIb IX X L	subctx	4072	-7	-55	-49	-15.84	1	2	-11.38	0	4	0	0
Cblm cortex L	Cblm V L	Cblm I IV V VI L	subctx	5016	-15	-50	-19	-3.13	3	0	3.63	3	0	2	0
Cblm cortex L	Cblm VI L	Cblm I IV V VI L	subctx	11192	-24	-59	-25	11.01	5	0	-4.5	3	1	2	0
Cblm cortex L	Cblm VIIIa L	Cblm VIIIa VIIb IX X L	subctx	6384	-25	-59	-52	12.39	6	0	-6.76	2	0	0	0
Cblm cortex L	Cblm VIIIb L	Cblm VIIIa VIIb IX X L	subctx	5368	-18	-51	-55	-9.83	2	0	-8.19	0	2	0	0
Cblm cortex L	Cblm VIIb L	Cblm CrusI CrusII VIIIb L	subctx	5552	-26	-67	-50	6	3	1	3.94	2	2	1	0
Cblm cortex L	Cblm X L	Cblm VIIIa VIIb IX X L	subctx	904	-21	-37	-45	1.63	1	0	-6.14	0	2	0	0
Cblm cortex R	Cblm CrusI R	Cblm CrusI CrusII VIIIb R	subctx	16136	37	-67	-33	-7	4	1	4.5	5	0	3	0
Cblm cortex R	Cblm CrusII R	Cblm CrusI CrusII VIIIb R	subctx	11832	26	-76	-42	-5.03	1	1	2.83	3	1	1	0
Cblm cortex R	Cblm I IV R	Cblm I IV V VI R	subctx	3672	10	-44	-17	4.44	3	0	3.08	2	1	1	0

Structure	Region	Parcel	Network	Volume	MNI Coordinates			Experienced Pain (Hot-Warm)			Imagined Pain (Imagine-Warm)			Pain Conjunction	
					X	Y	Z	T	Activated (n)	Deactivated (n)	T	Activated (n)	Deactivated (n)	Activated (n)	Deactivated (n)
Cblm cortex R	Cblm IX R	Cblm VIIIa VIIb IX X R	subctx	4416	7	-55	-49	-21.47	0	3	-5.83	0	2	0	1
Cblm cortex R	Cblm V R	Cblm I IV V VI R	subctx	5336	15	-51	-20	5.09	4	0	3.78	4	0	2	0
Cblm cortex R	Cblm VI R	Cblm I IV V VI R	subctx	9816	24	-59	-26	6.53	5	0	11.34	5	0	3	0
Cblm cortex R	Cblm VIIIa R	Cblm VIIIa VIIb IX X R	subctx	6464	24	-59	-53	7.66	4	0	6.85	3	1	1	0
Cblm cortex R	Cblm VIIIb R	Cblm VIIIa VIIb IX X R	subctx	5144	17	-52	-56	-4.5	2	0	-3.42	1	3	0	0
Cblm cortex R	Cblm VIIb R	Cblm CrusI CrusII VIIIb R	subctx	6216	27	-66	-51	6.63	4	0	3.97	4	0	1	0
Cblm cortex R	Cblm X R	Cblm VIIIa VIIb IX X R	subctx	920	22	-37	-46	-4.27	1	0	-5.19	1	2	0	0
Cblm vermis	Cblm Vermis CrusII	Cblm vermis	subctx	504	1	-75	-31	1.61	2	1	1.72	1	1	0	1
Cblm vermis	Cblm Vermis IX	Cblm vermis	subctx	976	-0	-57	-38	-2.83	2	0	-2.7	1	0	0	0
Cblm vermis	Cblm Vermis VI	Cblm vermis	subctx	2600	1	-71	-20	2.99	5	0	3.9	5	0	2	0
Cblm vermis	Cblm Vermis VIIIa	Cblm vermis	subctx	1464	0	-68	-37	-2.05	1	1	1.32	1	1	0	1
Cblm vermis	Cblm Vermis VIIIb	Cblm vermis	subctx	848	0	-65	-41	-1.39	3	1	-1.12	1	1	0	0
Cblm vermis	Cblm Vermis VIIb	Cblm vermis	subctx	216	0	-70	-31	-1.94	1	1	0.97	1	0	0	0
Cblm vermis	Cblm Vermis X	Cblm vermis	subctx	352	0	-49	-36	-5.35	0	0	-2.24	1	1	0	0
GP L	BG GPe L	GPe L	subctx	1648	-20	-4	-2	6.26	3	0	5.56	2	0	2	0
GP L	BG GPi L	GPi L	subctx	864	-18	-6	-4	5.23	3	0	4.71	2	0	1	0
GP R	BG GPe R	GPe R	subctx	1200	20	-2	-1	8.54	3	0	2.5	2	0	2	0
GP R	BG GPi R	GPi R	subctx	1056	19	-5	-4	5.56	2	0	4.68	1	0	1	0
Hippocampal Formation L	MTL Hipp CA1 L	Hipp L	subctx	2840	-29	-24	-14	-11.03	0	7	5.15	0	0	0	0
Hippocampal Formation L	MTL Hipp CA23 L	Hipp L	subctx	1344	-25	-20	-14	-9.45	0	6	7.04	1	0	0	0
Hippocampal Formation L	MTL Hipp DG L	Hipp L	subctx	872	-27	-26	-12	-5.11	0	5	2.64	0	0	0	0
Hippocampal Formation L	MTL Hipp Subiculum L	Subiculum L	subctx	1856	-21	-19	-19	-4.72	0	5	2.72	0	1	0	1
Hippocampal Formation R	MTL Hipp CA1 R	Hipp R	subctx	2960	28	-23	-14	-7.06	0	6	-6.93	1	0	0	0
Hippocampal Formation R	MTL Hipp CA23 R	Hipp R	subctx	1336	25	-18	-15	-4.98	0	6	5.94	2	0	0	0
Hippocampal Formation R	MTL Hipp DG R	Hipp R	subctx	560	29	-24	-13	-9.34	0	7	2.05	0	0	0	0
Hippocampal Formation R	MTL Hipp Subiculum R	Subiculum R	subctx	1928	22	-18	-19	-6.37	0	6	-4.81	0	1	0	0
Medulla	BStem RObPaMg	Medullary Raphe (Serotonergic)	subctx	584	-0	-37	-53	3.65	4	0	2.99	0	0	0	0
Medulla L	BStem OC L	Olivary complex L	subctx	832	-7	-37	-53	2.85	3	0	4.34	0	0	0	0
Medulla L	BStem PCRtA L	Medullary reticular formation L	subctx	112	-9	-39	-42	1.31	2	1	-1.94	0	0	0	0

Structure	Region	Parcel	Network	Volume	MNI Coordinates X	Y	Z	Experienced Pain (Hot-Warm) T	Activated (n)	Deactivated (n)	Imagined Pain (Imagine-Warm) T	Activated (n)	Deactivated (n)	Pain Conjunction Activated (n)	Deactivated (n)
Medulla L	BStem Shen Med L	Shen Med L	subctx	1952	-4	-41	-59	-5.54	2	2	3.33	0	0	0	0
Medulla L	BStem VSM L	Cranial nuclei L	subctx	208	-3	-45	-54	2.28	2	0	2.22	1	0	0	0
Medulla L	BStem Ve L	Cranial nuclei L	subctx	456	-7	-42	-39	-4.99	1	1	-3.08	0	0	0	0
Medulla L	BStem iMRt L	Medullary reticular formation L	subctx	632	-5	-44	-59	2.29	2	0	3.48	0	0	0	0
Medulla L	BStem sMRt L	Medullary reticular formation L	subctx	152	-6	-42	-47	-2.33	3	1	1.2	0	0	0	0
Medulla R	BStem OC R	Olivary complex R	subctx	784	6	-36	-53	3.01	5	0	4.18	0	0	0	0
Medulla R	BStem PCRtA R	Medullary reticular formation R	subctx	168	9	-38	-42	2.43	2	0	1.73	0	0	0	0
Medulla R	BStem Shen Med R	Shen Med R	subctx	2104	5	-41	-60	-5.16	1	0	8.21	0	0	0	0
Medulla R	BStem VSM R	Cranial nuclei R	subctx	160	2	-44	-54	2.3	2	0	3.15	0	0	0	0
Medulla R	BStem Ve R	Cranial nuclei R	subctx	344	6	-42	-40	-4.88	0	1	-1.58	0	1	0	1
Medulla R	BStem iMRt R	Medullary reticular formation R	subctx	752	4	-44	-59	-3.8	2	0	6.3	0	0	0	0
Medulla R	BStem sMRt R	Medullary reticular formation R	subctx	160	6	-42	-48	-3.09	4	1	-3.55	0	1	0	1
Midbrain	BStem CLi RLi	Rostral Raphe (Serotonergic)	subctx	264	-1	-24	-9	6.83	5	0	1.29	0	0	0	0
Midbrain	BStem DR B7	Rostral Raphe (Serotonergic)	subctx	112	-0	-34	-19	1.86	2	0	1.03	0	0	0	0
Midbrain	BStem MnR	Rostral Raphe (Serotonergic)	subctx	120	0	-31	-21	2.18	2	0	-1.71	0	0	0	0
Midbrain	BStem PAG	PAG	subctx	712	0	-32	-10	3.06	4	0	2.25	2	2	0	0
Midbrain L	BStem IC L	Tectum L	subctx	240	-6	-38	-10	-4.03	0	0	1.92	2	1	0	0
Midbrain L	BStem MiTg PBG L	Tectum L	subctx	96	-9	-32	-10	1.72	2	0	1.46	0	0	0	0
Midbrain L	BStem RN L	RN L	subctx	416	-5	-19	-9	4.78	3	0	1.18	0	0	0	0
Midbrain L	BStem SC L	Tectum L	subctx	248	-5	-33	-4	-2.25	1	0	-0.78	1	2	0	0
Midbrain L	BStem SN L	VTA PBP SN L	subctx	552	-9	-17	-13	4.62	3	0	2.53	1	0	1	0
Midbrain L	BStem STH L	STH L	subctx	224	-10	-11	-7	4.37	2	0	2.88	1	0	1	0
Midbrain L	BStem Shen Midb Lc	Shen Midb Lc	subctx	4848	-8	-19	-16	-5.16	5	0	6.81	2	0	0	0
Midbrain L	BStem Shen Midb Lrd	Shen Midb Lrd	subctx	2264	-13	-21	-6	9.15	2	0	9.66	3	0	1	0
Midbrain L	BStem VTA PBP L	VTA PBP SN L	subctx	160	-7	-18	-12	3.99	3	0	1.47	1	0	1	0
Midbrain L	BStem isRt L	Rostral reticular formation L	subctx	160	-6	-29	-8	2.64	1	0	-0.61	1	2	0	0
Midbrain L	BStem mRt L	Rostral reticular formation L	subctx	688	-8	-25	-3	5.47	5	0	1.86	2	1	1	0
Midbrain R	BStem IC R	Tectum R	subctx	296	5	-37	-9	-4.24	0	0	2.78	1	2	0	0
Midbrain R	BStem MiTg PBG R	Tectum R	subctx	80	9	-31	-10	1.77	3	0	-2.71	0	0	0	0

Structure	Region	Parcel	Network	Volume	MNI Coordinates			Experienced Pain (Hot-Warm)			Imagined Pain (Imagine-Warm)			Pain Conjunction	
					X	Y	Z	T	Activated (n)	Deactivated (n)	T	Activated (n)	Deactivated (n)	Activated (n)	Deactivated (n)
Midbrain R	BStem RN R	RN R	subctx	392	5	-19	-9	4.49	4	0	-1.96	1	0	1	0
Midbrain R	BStem SC R	Tectum R	subctx	192	4	-33	-4	-4.46	1	0	-2.18	1	3	0	0
Midbrain R	BStem SN R	VTA PBP SN R	subctx	560	10	-17	-13	4.58	5	0	4.15	1	0	1	0
Midbrain R	BStem STH R	STH R	subctx	232	11	-11	-7	5.44	4	0	4.46	1	0	1	0
Midbrain R	BStem Shen Midb Rcd	Shen Midb Rcd	subctx	5392	9	-21	-16	5.91	3	0	4.74	1	0	0	0
Midbrain R	BStem Shen Midb Rrd	Shen Midb Rrd	subctx	1936	13	-21	-5	5.32	4	0	3.99	2	0	1	0
Midbrain R	BStem VTA PBP R	VTA PBP SN R	subctx	152	7	-18	-13	4.14	5	0	1.19	2	0	2	0
Midbrain R	BStem isRt R	Rostral reticular formation R	subctx	120	6	-28	-8	3.21	4	0	-1.6	1	1	0	0
Midbrain R	BStem mRt R	Rostral reticular formation R	subctx	720	8	-24	-4	3.47	6	0	-2.09	3	1	1	0
PUT L	BG PUT DA L	aPUT L	subctx	1912	-25	6	0	19.35	6	0	4.14	3	1	3	0
PUT L	BG PUT DP L	pPUT L	subctx	1952	-27	-2	8	7.96	4	0	5.4	3	0	2	0
PUT L	BG PUT VA L	aPUT L	subctx	2504	-21	10	-7	10.33	4	2	5.84	4	0	3	0
PUT L	BG PUT VP L	pPUT L	subctx	2736	-31	-11	-1	8.65	3	0	6.22	3	0	2	0
PUT R	BG PUT DA R	aPUT R	subctx	2048	26	7	-0	6.86	5	0	3.02	4	0	4	0
PUT R	BG PUT DP R	pPUT R	subctx	1584	27	-2	6	6.17	4	0	2.85	3	0	3	0
PUT R	BG PUT VA R	aPUT R	subctx	2624	20	12	-7	8.92	4	0	5.49	3	0	3	0
PUT R	BG PUT VP R	pPUT R	subctx	2368	31	-10	-2	14.22	5	0	6.04	3	0	2	0
Pons L	BStem LC+ L	LC+ L	subctx	176	-7	-37	-35	1.49	2	0	3.64	0	0	0	0
Pons L	BStem LDTg CGPn L	Cholinergic nuclei L	subctx	488	-2	-40	-33	2.7	1	1	2.86	0	0	0	0
Pons L	BStem LPB L	Parabrachial nuclei L	subctx	288	-8	-38	-21	1.45	1	0	3.9	1	0	0	0
Pons L	BStem MPB L	Parabrachial nuclei L	subctx	216	-6	-37	-24	1.24	2	0	2.24	0	0	0	0
Pons L	BStem PTg L	Cholinergic nuclei L	subctx	144	-8	-30	-14	2.18	4	0	1.23	0	0	0	0
Pons L	BStem PnO PnC L	L PnO PnC L	subctx	680	-3	-35	-33	-5.88	2	0	-1.79	0	1	0	0
Pons L	BStem Shen Pons Lcd	Shen Pons Lcd	subctx	2520	-9	-32	-40	-8.96	1	3	-3.23	0	1	0	0
Pons L	BStem Shen Pons Lrd	Shen Pons Lrd	subctx	1744	-9	-36	-28	-7.36	1	0	13.53	1	0	0	0
Pons L	BStem Shen Pons Lv	Shen Pons Lv	subctx	4568	-7	-21	-35	-14.04	0	3	3.13	0	0	0	0
Pons R	BStem LC+ R	LC+ R	subctx	176	7	-38	-35	2.35	3	0	-1.59	1	0	1	0
Pons R	BStem LDTg CGPn R	Cholinergic nuclei R	subctx	440	2	-40	-31	1.66	2	1	-2.14	1	1	1	1
Pons R	BStem LPB R	Parabrachial nuclei R	subctx	328	7	-38	-21	1.31	2	0	1.87	1	0	0	0
Pons R	BStem MPB R	Parabrachial nuclei R	subctx	216	5	-38	-24	1.39	2	0	2.05	1	0	0	0
Pons R	BStem PTg R	Cholinergic nuclei R	subctx	160	7	-30	-15	3.01	4	0	2.25	0	0	0	0
Pons R	BStem PnO PnC R	R PnO PnC R	subctx	616	3	-35	-33	-2.39	4	0	-3.99	1	0	1	0

					MNI Coordinates			Experienced Pain (Hot-Warm)			Imagined Pain (Imagine-Warm)			Pain Conjunction	
Structure	Region	Parcel	Network	Volume	X	Y	Z	T	Activated (n)	Deactivated (n)	T	Activated (n)	Deactivated (n)	Activated (n)	Deactivated (n)
Pons R	BStem Shen Pons Rcd	Shen Pons Rcd	subctx	2488	9	-36	-36	-11.64	2	0	-3.94	0	0	0	0
Pons R	BStem Shen Pons Rcv	Shen Pons Rcv	subctx	1920	6	-24	-43	-5.24	0	2	-1.98	0	0	0	0
Pons R	BStem Shen Pons Rrv	Shen Pons Rrv	subctx	4224	10	-20	-30	-5.74	0	2	-4.19	0	2	0	0
Thal Anterior L	Thal AV L	Thal anterior L	subctx	760	-4	-4	10	4.62	4	0	1.9	2	0	2	0
Thal Anterior R	Thal AV R	Thal anterior R	subctx	872	4	-3	9	5.32	4	0	1.98	0	0	0	0
Thal Intralaminar L	Thal CL L	Thal Intralaminar-anterodorsal L	subctx	160	-2	-11	13	3.65	4	0	1.5	2	0	2	0
Thal Intralaminar L	Thal cIL L	Thal Intralaminar-posterior L	subctx	448	-10	-20	1	3.35	6	0	3.36	2	0	2	0
Thal Intralaminar L	Thal rIL L	Thal Intralaminar-antoventral L	subctx	216	-3	-10	-1	3.29	5	0	1.17	0	0	0	0
Thal Intralaminar R	Thal CL R	Thal Intralaminar-anterodorsal R	subctx	112	4	-12	13	3.5	6	0	0.43	1	1	1	0
Thal Intralaminar R	Thal cIL R	Thal Intralaminar-posterior R	subctx	432	10	-19	1	6.06	6	0	2.92	3	0	2	0
Thal Intralaminar R	Thal rIL R	Thal Intralaminar-antoventral R	subctx	208	4	-9	-1	3.12	3	0	1.67	0	0	0	0
Thal Lateral L	Thal LD L	Thal dorsal L	subctx	336	-5	-15	18	5.15	2	0	1.12	0	0	0	0
Thal Lateral L	Thal LP L	Thal dorsal L	subctx	392	-10	-16	18	3.79	3	0	1.83	1	0	1	0
Thal Lateral R	Thal LD R	Thal dorsal R	subctx	344	5	-15	17	4.35	4	0	-1.69	0	1	0	0
Thal Lateral R	Thal LP R	Thal dorsal R	subctx	440	11	-15	18	4.92	5	0	1.45	1	0	0	0
Thal Medial L	Thal MD L	Thal Medial L	subctx	1480	-4	-15	6	3.62	4	0	2.81	2	0	2	0
Thal Medial R	Thal MD R	Thal Medial R	subctx	1480	5	-14	6	3.72	5	0	1.91	2	1	2	0
Thal Posterior L	Thal LGN L	Thal LGN L	subctx	848	-24	-27	-3	4.36	1	1	4.83	0	3	0	0
Thal Posterior L	Thal MGN L	Thal MGN L	subctx	424	-16	-29	-3	-2.96	1	1	2.96	0	1	0	0
Thal Posterior L	Thal PuA L	Thal Pulvinar L	subctx	376	-11	-21	11	3.06	4	0	2.96	1	0	1	0
Thal Posterior L	Thal PuL L	Thal Pulvinar L	subctx	728	-19	-27	7	-4.02	2	0	3.39	1	0	0	0
Thal Posterior L	Thal PuM L	Thal Pulvinar L	subctx	3272	-11	-29	9	-6.2	3	0	14.5	1	0	0	0
Thal Posterior R	Thal LGN R	Thal LGN R	subctx	752	24	-26	-3	2.98	2	0	3.67	1	2	0	0
Thal Posterior R	Thal MGN R	Thal MGN R	subctx	408	16	-27	-2	-5.22	1	1	-2.57	1	1	1	0
Thal Posterior R	Thal PuA R	Thal Pulvinar R	subctx	408	12	-20	11	3.65	5	0	1.63	1	0	1	0
Thal Posterior R	Thal PuL R	Thal Pulvinar R	subctx	712	19	-25	7	-3.95	3	1	2.57	0	1	0	0
Thal Posterior R	Thal PuM R	Thal Pulvinar R	subctx	3096	12	-28	9	-6.02	4	0	-2.68	1	0	0	0
Thal Ventral L	Thal VA L	Thal Lateral-rostral L	subctx	864	-8	-4	4	4.91	4	0	1.69	3	0	3	0
Thal Ventral L	Thal VL L	Thal Lateral-rostral L	subctx	2016	-13	-11	9	6.93	5	0	3.12	3	0	3	0
Thal Ventral L	Thal VPL VPM L	Thal Lateral-caudal L	subctx	1432	-18	-20	5	4.26	5	0	4.16	4	0	3	0
Thal Ventral R	Thal VA R	Thal Lateral-rostral R	subctx	864	9	-3	4	10.16	6	0	2.94	2	0	2	0

Structure	Region	Parcel	Network	Volume	MNI Coordinates			Experienced Pain (Hot-Warm)			Imagined Pain (Imagine-Warm)			Pain Conjunction	
					X	Y	Z	T	Activated (n)	Deactivated (n)	T	Activated (n)	Deactivated (n)	Activated (n)	Deactivated (n)
Thal Ventral R	Thal VL R	Thal Lateral-rostral R	subctx	2040	14	-10	9	7.03	5	0	2.52	2	1	2	0
Thal Ventral R	Thal VPL VPM R	Thal Lateral-caudal R	subctx	1368	18	-19	5	5.3	5	0	2.92	3	0	2	0
VStriatum L	BG BST SLEA L	VStriatum L	subctx	608	-7	4	-1	-2.55	1	3	-5.23	2	1	1	0
VStriatum L	BG NAc L	VStriatum L	subctx	2576	-8	13	-7	-4.28	0	4	-9.42	0	2	0	2
VStriatum R	BG BST SLEA R	VStriatum R	subctx	816	7	5	-0	5.32	3	2	-3.97	2	1	2	0
VStriatum R	BG NAc R	VStriatum R	subctx	2552	7	14	-7	-4.49	0	3	-6.73	0	1	0	1
auditory association cortex L	Ctx A4 L	auditory transition area L	Cortex_SomatomotorB	4032	-63	-23	10	5.82	5	3	-6.89	2	2	1	2
auditory association cortex L	Ctx A5 L	auditory transition area L	Cortex_Temporal_Parietal	3704	-63	-13	-1	-5.26	5	3	-4.46	2	3	2	1
auditory association cortex L	Ctx STGa L	auditory transition area L	Cortex_Temporal_Parietal	3064	-51	13	-14	8.06	4	1	3	2	3	1	2
auditory association cortex L	Ctx STSda L	anterior AAC L	Cortex_Default_ModeB	2448	-55	-7	-9	-5.64	1	3	-3.87	0	6	0	4
auditory association cortex L	Ctx STSdp L	posterior AAC L	Cortex_Default_ModeB	2656	-52	-31	2	-5.15	0	4	-4.46	2	3	0	2
auditory association cortex L	Ctx STSva L	anterior AAC L	Cortex_Default_ModeB	2384	-51	-10	-15	-7.53	0	5	-3.82	1	3	0	2
auditory association cortex L	Ctx STSvp L	posterior AAC L	Cortex_Default_ModeB	4080	-54	-34	-4	-3.54	0	4	-5.44	1	4	0	1
auditory association cortex L	Ctx TA2 L	auditory transition area L	Cortex_Temporal_Parietal	1544	-50	0	-5	11.99	9	1	-1.68	2	2	2	0
auditory association cortex R	Ctx A4 R	auditory transition area R	Cortex_SomatomotorB	3864	65	-14	7	8.28	5	3	-9.08	1	3	1	2
auditory association cortex R	Ctx A5 R	auditory transition area R	Cortex_Temporal_Parietal	4616	63	-12	-2	-8.57	2	4	-4.95	0	4	2	1
auditory association cortex R	Ctx STGa R	auditory transition area R	Cortex_Temporal_Parietal	2688	51	16	-17	-10.3	7	1	-4.66	2	1	2	1
auditory association cortex R	Ctx STSda R	anterior AAC R	Cortex_Temporal_Parietal	3160	52	-2	-14	-11.18	0	5	-12.89	0	4	0	3
auditory association cortex R	Ctx STSdp R	posterior AAC R	Cortex_Temporal_Parietal	3632	49	-27	-2	-4.87	2	1	-3.87	1	3	0	1
auditory association cortex R	Ctx STSva R	anterior AAC R	Cortex_Temporal_Parietal	2384	54	-10	-17	-6.91	0	5	-6.04	0	6	0	4
auditory association cortex R	Ctx STSvp R	posterior AAC R	Cortex_Default_ModeB	3240	57	-30	-5	-4.08	2	2	-5.43	1	4	0	2
auditory association cortex R	Ctx TA2 R	auditory transition area R	Cortex_SomatomotorB	2040	51	2	-5	6.74	7	0	-9.66	3	2	3	0
auditory early L	Ctx A1 L	A1 L	Cortex_SomatomotorB	1448	-43	-24	12	6.76	7	1	-3.35	0	2	0	1
auditory early L	Ctx LBelt L	auditory+ L	Cortex_SomatomotorB	1360	-44	-27	8	3.39	2	2	-3.63	0	2	0	1
auditory early L	Ctx MBelt L	auditory+ L	Cortex_SomatomotorB	1872	-45	-16	4	6.44	7	2	4.61	0	2	0	0
auditory early L	Ctx PBelt L	auditory+ L	Cortex_SomatomotorB	2504	-53	-26	9	4.14	4	3	-4.05	0	2	0	1
auditory early L	Ctx RI L	auditory+ L	Cortex_SomatomotorB	2488	-39	-34	20	-3.47	2	3	8.13	0	2	0	2
auditory early R	Ctx A1 R	A1 R	Cortex_SomatomotorB	888	43	-21	11	5.89	7	1	-2.57	0	2	0	1
auditory early R	Ctx LBelt R	auditory+ R	Cortex_SomatomotorB	1624	48	-24	10	4.25	2	2	-3.07	0	2	0	1
auditory early R	Ctx MBelt R	auditory+ R	Cortex_SomatomotorB	1440	46	-15	5	13.86	8	0	-3.5	0	3	0	1
auditory early R	Ctx PBelt R	auditory+ R	Cortex_SomatomotorB	1856	57	-18	8	7.69	6	2	-2.74	0	3	0	1

					MNI Coordinates			Experienced Pain (Hot-Warm)			Imagined Pain (Imagine-Warm)			Pain Conjunction	
Structure	Region	Parcel	Network	Volume	X	Y	Z	T	Activated (n)	Deactivated (n)	T	Activated (n)	Deactivated (n)	Activated (n)	Deactivated (n)
auditory early R	Ctx RI R	auditory+ R	Cortex_SomatomotorB	2056	40	-30	20	4.61	3	3	-4.19	0	3	0	2
cingulate ACC mPFC L	Ctx 25 L	infralimbic L	Cortex_Default_ModeA	2304	-5	24	-11	-10.86	0	5	-5	0	2	0	2
cingulate ACC mPFC L	Ctx 33pr L	midcingulate L	Cortex_Ventral_AttentionB	1752	-4	11	28	7.83	4	1	-2.62	0	3	0	1
cingulate ACC mPFC L	Ctx 8BM L	dmPFC L	Cortex_Fronto_ParietalB	5048	-6	31	45	4.6	2	2	2.63	2	3	1	0
cingulate ACC mPFC L	Ctx 9m L	dmPFC L	Cortex_Default_ModeB	8768	-6	53	26	-5.26	0	3	-3.06	1	4	0	1
cingulate ACC mPFC L	Ctx a24 L	vmPFC L	Cortex_Default_ModeA	3192	-5	41	-2	-3.89	0	4	-3.34	0	1	0	1
cingulate ACC mPFC L	Ctx a24pr L	midcingulate L	Cortex_Ventral_AttentionB	1856	-5	17	32	9.97	6	0	4.74	1	1	1	0
cingulate ACC mPFC L	Ctx a32pr L	midcingulate L	Cortex_Ventral_AttentionB	2360	-10	29	30	4.12	3	1	-3.12	1	2	1	0
cingulate ACC mPFC L	Ctx d32 L	dmPFC L	Cortex_Default_ModeA	3152	-8	40	27	-6.1	0	3	-3.4	1	5	0	1
cingulate ACC mPFC L	Ctx p24 L	midcingulate L	Cortex_Default_ModeA	3352	-5	36	15	2.61	2	2	-3.19	0	2	0	1
cingulate ACC mPFC L	Ctx p24pr L	midcingulate L	Cortex_Ventral_AttentionA	1976	-4	-4	40	7.51	6	0	2.58	2	1	1	0
cingulate ACC mPFC L	Ctx p32 L	vmPFC L	Cortex_Default_ModeA	1664	-13	49	1	-4.63	0	2	-4.88	1	2	0	1
cingulate ACC mPFC L	Ctx p32pr L	midcingulate L	Cortex_Ventral_AttentionA	2920	-9	14	40	8.06	6	0	3.16	4	1	2	0
cingulate ACC mPFC L	Ctx s32 L	vmPFC L	Cortex_Default_ModeA	880	-8	35	-12	-3.9	0	4	-12.09	0	1	0	1
cingulate ACC mPFC R	Ctx 25 R	infralimbic R	Cortex_Limbic	1904	3	23	-10	-14.6	0	6	-3.19	0	3	0	3
cingulate ACC mPFC R	Ctx 33pr R	midcingulate R	Cortex_Ventral_AttentionB	2168	5	12	27	5.71	4	0	-2.83	0	2	0	0
cingulate ACC mPFC R	Ctx 8BM R	dmPFC R	Cortex_Fronto_ParietalB	6232	7	27	48	5.39	4	0	-11.8	1	3	1	0
cingulate ACC mPFC R	Ctx 9m R	dmPFC R	Cortex_Default_ModeA	10400	8	53	23	-8.08	0	5	-7.72	0	5	0	1
cingulate ACC mPFC R	Ctx a24 R	vmPFC R	Cortex_Default_ModeA	2936	6	38	0	-4.29	1	4	-3.55	1	2	1	1
cingulate ACC mPFC R	Ctx a24pr R	midcingulate R	Cortex_Ventral_AttentionB	2024	5	17	34	4.99	7	0	3.39	1	2	2	0
cingulate ACC mPFC R	Ctx a32pr R	midcingulate R	Cortex_Ventral_AttentionB	1696	12	26	32	-5.16	4	0	5.07	1	1	1	0
cingulate ACC mPFC R	Ctx d32 R	dmPFC R	Cortex_Ventral_AttentionB	3848	10	38	28	-3.57	2	2	-11.62	0	4	0	0
cingulate ACC mPFC R	Ctx p24 R	midcingulate R	Cortex_Ventral_AttentionB	3048	6	36	17	-2.6	3	2	-2.9	0	3	1	1
cingulate ACC mPFC R	Ctx p24pr R	midcingulate R	Cortex_Ventral_AttentionA	2584	6	-4	41	6.24	6	0	3.28	1	2	1	0
cingulate ACC mPFC R	Ctx p32 R	vmPFC R	Cortex_Default_ModeA	2040	11	49	-1	-4.3	1	4	-4.45	0	2	0	0
cingulate ACC mPFC R	Ctx p32pr R	midcingulate R	Cortex_Ventral_AttentionB	2632	12	13	41	4.68	6	0	2.95	3	0	2	0
cingulate ACC mPFC R	Ctx s32 R	vmPFC R	Cortex_Default_ModeA	1296	6	36	-13	-7.48	0	5	-5.13	0	3	0	2

Structure	Region	Parcel	Network	Volume	MNI Coordinates			Experienced Pain (Hot-Warm)			Imagined Pain (Imagine-Warm)			Pain Conjunction	
					X	Y	Z	T	Activated (n)	Deactivated (n)	T	Activated (n)	Deactivated (n)	Activated (n)	Deactivated (n)
cingulate dlPFC L	Ctx 46 L	BA46 L	Cortex_Ventral_AttentionB	7480	-37	37	32	10.43	6	0	4.69	4	2	5	0
cingulate dlPFC L	Ctx 8Ad L	Superior lateral BA8 L	Cortex_Default_ModeA	6368	-24	26	43	-12.18	0	6	-6.17	0	4	0	2
cingulate dlPFC L	Ctx 8Av L	Inferior lateral BA8 L	Cortex_Default_ModeB	5984	-38	17	52	-4.31	0	4	-3.63	2	1	0	0
cingulate dlPFC L	Ctx 8BL L	Superior lateral BA8 L	Cortex_Default_ModeB	4520	-12	35	55	-5.82	0	4	-2.96	1	1	0	0
cingulate dlPFC L	Ctx 8C L	Inferior lateral BA8 L	Cortex_Fronto_ParietalA	7160	-41	14	38	-5	1	3	5.12	1	1	1	0
cingulate dlPFC L	Ctx 9 46d L	BA46 L	Cortex_Ventral_AttentionB	6784	-29	45	23	5.04	4	1	6.43	5	0	3	0
cingulate dlPFC L	Ctx 9a L	BA9 L	Cortex_Default_ModeB	5032	-20	55	27	-6	1	5	-2.27	4	1	2	2
cingulate dlPFC L	Ctx 9p L	BA9 L	Cortex_Default_ModeB	4520	-20	45	40	-8.44	4	2	-3	2	2	2	1
cingulate dlPFC L	Ctx SFL L	Superior lateral BA8 L	Cortex_Default_ModeB	5616	-8	16	65	-7.79	2	2	-3.05	4	2	3	0
cingulate dlPFC L	Ctx a9 46v L	BA46 L	Cortex_Ventral_AttentionB	4632	-38	50	10	3.53	4	2	5.47	3	1	3	0
cingulate dlPFC L	Ctx i6 8 L	Inferior lateral BA8 L	Cortex_Dorsal_AttentionB	2496	-32	5	60	-3.66	0	3	2.38	3	0	0	0
cingulate dlPFC L	Ctx p9 46v L	BA46 L	Cortex_Fronto_ParietalA	4432	-44	28	28	4.08	3	3	3.66	5	0	3	1
cingulate dlPFC L	Ctx s6 8 L	Superior lateral BA8 L	Cortex_Default_ModeA	2088	-23	22	58	-4.16	0	4	-2.37	2	1	0	1
cingulate dlPFC R	Ctx 46 R	BA46 R	Cortex_Ventral_AttentionB	8176	36	38	31	9.74	6	0	-5.08	1	3	2	0
cingulate dlPFC R	Ctx 8Ad R	Superior lateral BA8 R	Cortex_Default_ModeA	5296	24	27	45	-7.8	0	5	-13.61	0	4	0	3
cingulate dlPFC R	Ctx 8Av R	Inferior lateral BA8 R	Cortex_Fronto_ParietalB	7128	40	19	49	4.65	1	3	-8.59	0	4	1	1
cingulate dlPFC R	Ctx 8BL R	Superior lateral BA8 R	Cortex_Default_ModeB	7312	13	38	53	-6.31	1	4	-10.95	0	5	0	2
cingulate dlPFC R	Ctx 8C R	Inferior lateral BA8 R	Cortex_Fronto_ParietalA	5992	38	16	36	-5.29	2	3	-9.34	0	2	0	1
cingulate dlPFC R	Ctx 9 46d R	BA46 R	Cortex_Ventral_AttentionB	7896	29	48	24	8.95	6	0	-4.32	3	2	2	0
cingulate dlPFC R	Ctx 9a R	BA9 R	Cortex_Default_ModeA	4088	20	61	22	-6.94	0	3	-4.58	1	3	0	0
cingulate dlPFC R	Ctx 9p R	BA9 R	Cortex_Default_ModeB	3936	20	48	36	-5.14	4	1	-3.07	3	2	3	0
cingulate dlPFC R	Ctx SFL R	Superior lateral BA8 R	Cortex_Default_ModeB	3792	8	13	67	2.92	3	3	-4.13	5	1	3	0
cingulate dlPFC R	Ctx a9 46v R	BA46 R	Cortex_Ventral_AttentionB	3664	40	52	10	5.15	6	2	-7.57	2	2	2	1
cingulate dlPFC R	Ctx i6 8 R	Inferior lateral BA8 R	Cortex_Fronto_ParietalB	2384	35	9	60	-4.27	2	2	-4.66	0	3	0	1
cingulate dlPFC R	Ctx p9 46v R	BA46 R	Cortex_Fronto_ParietalA	5560	46	31	29	8.61	3	1	-11.59	0	4	0	0
cingulate dlPFC R	Ctx s6 8 R	Superior lateral BA8 R	Cortex_Default_ModeA	3024	22	18	60	-3.43	0	4	-2.91	0	4	0	1
cingulate posterior L	Ctx 23c L	anterior precuneus L	Cortex_Ventral_AttentionA	2856	-12	-31	42	-7.47	3	3	-5.4	1	3	0	2
cingulate posterior L	Ctx 23d L	anterior precuneus L	Cortex_Default_ModeA	1728	-3	-22	39	5.1	2	2	-9.92	0	3	0	1
cingulate posterior L	Ctx 31a L	anterior precuneus L	Cortex_Default_ModeA	1912	-4	-38	44	-7.57	1	4	-6.04	1	4	0	2
cingulate posterior L	Ctx 31pd L	precuneus L	Cortex_Default_ModeA	2144	-10	-52	36	-15.2	0	5	-6.02	0	3	0	1
cingulate posterior L	Ctx 31pv L	precuneus L	Cortex_Default_ModeA	1328	-11	-46	32	-12.21	0	4	-4.26	0	3	0	1
cingulate posterior L	Ctx 7m L	precuneus L	Cortex_Default_ModeA	2952	-4	-62	37	-19.48	1	4	-3.91	0	4	0	2
cingulate posterior L	Ctx DVT L	parietal occipital sulcus L	Cortex_Visual_Peripheral	2944	-20	-71	32	-8.19	0	4	-5.4	0	2	0	1

					MNI Coordinates			Experienced Pain (Hot-Warm)			Imagined Pain (Imagine-Warm)			Pain Conjunction	
Structure	Region	Parcel	Network	Volume	X	Y	Z	T	Activated (n)	Deactivated (n)	T	Activated (n)	Deactivated (n)	Activated (n)	Deactivated (n)
cingulate posterior L	Ctx PCV L	PCV L	Cortex_Default_ModeA	3280	-6	-51	51	-12.21	1	3	-6.75	1	3	0	2
cingulate posterior L	Ctx POS1 L	parietal occipital sulcus L	Cortex_Default_ModeC	3504	-13	-57	16	-11.73	1	5	-5.84	0	2	0	1
cingulate posterior L	Ctx POS2 L	parietal occipital sulcus L	Cortex_Fronto_ParietalC	5736	-10	-71	40	11.11	5	0	-6.73	2	1	1	1
cingulate posterior L	Ctx ProS L	parietal occipital sulcus L	Cortex_Visual_Peripheral	2944	-24	-55	7	-4.07	1	3	3.88	2	2	0	1
cingulate posterior L	Ctx RSC L	RSC L	Cortex_Default_ModeC	5144	-5	-38	18	-14.66	2	2	-7.98	1	2	0	1
cingulate posterior L	Ctx d23ab L	precuneus L	Cortex_Default_ModeA	1960	-4	-43	31	-5.24	1	4	-5.02	0	4	0	1
cingulate posterior L	Ctx v23ab L	precuneus L	Cortex_Default_ModeA	1848	-4	-58	18	-6.18	0	4	-3.81	0	3	0	2
cingulate posterior R	Ctx 23c R	anterior precuneus R	Cortex_Ventral_AttentionA	3184	12	-33	44	-5.07	2	3	-3.96	1	3	0	1
cingulate posterior R	Ctx 23d R	anterior precuneus R	Cortex_Default_ModeA	2064	4	-23	40	3.13	3	1	-3.55	0	3	0	1
cingulate posterior R	Ctx 31a R	anterior precuneus R	Cortex_Default_ModeA	1760	7	-40	43	-14.54	0	4	-5.16	1	4	0	2
cingulate posterior R	Ctx 31pd R	precuneus R	Cortex_Default_ModeA	1416	12	-52	37	-10.59	1	3	-3.61	0	4	0	1
cingulate posterior R	Ctx 31pv R	precuneus R	Cortex_Default_ModeA	1448	12	-44	33	-4.33	1	3	-3.29	0	4	0	1
cingulate posterior R	Ctx 7m R	precuneus R	Cortex_Default_ModeA	3496	5	-62	37	-13.69	1	5	-4.18	0	4	0	3
cingulate posterior R	Ctx DVT R	parietal occipital sulcus R	Cortex_Fronto_ParietalC	3312	23	-66	31	-3.06	1	3	-4.69	0	4	0	1
cingulate posterior R	Ctx PCV R	PCV R	Cortex_Fronto_ParietalC	3592	7	-52	52	-10.32	0	3	-4.83	1	3	0	2
cingulate posterior R	Ctx POS1 R	parietal occipital sulcus R	Cortex_Default_ModeC	3392	15	-55	18	-9.27	1	4	-11.57	0	4	0	2
cingulate posterior R	Ctx POS2 R	parietal occipital sulcus R	Cortex_Fronto_ParietalC	6144	13	-68	40	4.25	4	2	-4.88	0	3	0	1
cingulate posterior R	Ctx ProS R	parietal occipital sulcus R	Cortex_Visual_Peripheral	2120	25	-48	6	-7.37	1	2	4.98	2	2	0	1
cingulate posterior R	Ctx RSC R	RSC R	Cortex_Default_ModeA	5368	6	-37	19	15.72	3	2	-10.67	0	5	0	1
cingulate posterior R	Ctx d23ab R	precuneus R	Cortex_Default_ModeA	1424	5	-41	33	10.7	1	3	-3.69	0	4	0	1
cingulate posterior R	Ctx v23ab R	precuneus R	Cortex_Default_ModeA	1880	5	-54	20	-6.45	0	4	-4.27	0	4	0	2
cingulate ventral frontal L	Ctx 10d L	BA10 L	Cortex_Default_ModeA	6120	-11	64	10	-7.05	1	4	-5.31	0	2	0	1
cingulate ventral frontal L	Ctx 10pp L	BA10 L	Cortex_Limbic	2704	-15	61	-13	-3.69	2	2	3.9	1	0	0	0
cingulate ventral frontal L	Ctx 10r L	BA10 L	Cortex_Default_ModeA	2568	-7	52	-7	-4.9	0	6	-3.06	0	2	0	1
cingulate ventral frontal L	Ctx 10v L	BA10 L	Cortex_Limbic	6248	-5	52	-16	-6.99	0	7	-7.82	0	1	0	1
cingulate ventral frontal L	Ctx 11l L	BA11 L	Cortex_Ventral_AttentionB	4712	-27	47	-13	7.6	4	2	3.2	2	0	1	0
cingulate ventral frontal L	Ctx 13l L	BA13 L	Cortex_Limbic	3648	-23	30	-18	3.5	1	3	5.9	4	0	0	0
cingulate ventral frontal L	Ctx 47l L	BA47 L	Cortex_Default_ModeB	3600	-46	30	-10	-3.74	2	3	3.17	2	1	1	1
cingulate ventral frontal L	Ctx 47m L	BA47 L	Cortex_Default_ModeB	912	-36	33	-14	-2.13	0	2	1.95	2	0	0	0
cingulate ventral frontal L	Ctx 47s L	BA47 L	Cortex_Default_ModeB	4056	-33	22	-20	-3.62	1	3	3.65	1	1	0	0
cingulate ventral frontal L	Ctx OFC L	OFC L	Cortex_Limbic	6640	-12	31	-21	-6.44	0	3	-8.45	0	1	0	1

Structure	Region	Parcel	Network	Volume	MNI Coordinates			Experienced Pain (Hot-Warm)			Imagined Pain (Imagine-Warm)			Pain Conjunction	
					X	Y	Z	T	Activated (n)	Deactivated (n)	T	Activated (n)	Deactivated (n)	Activated (n)	Deactivated (n)
cingulate ventral frontal L	Ctx a10p L	BA10 L	Cortex_Fronto_ParietalB	4288	-26	52	-3	-4.38	1	1	-4.3	1	2	0	0
cingulate ventral frontal L	Ctx a47r L	BA47 L	Cortex_Fronto_ParietalB	5272	-39	46	-9	-3.31	1	3	-2.37	3	2	0	1
cingulate ventral frontal L	Ctx p10p L	BA10 L	Cortex_Fronto_ParietalB	3544	-24	60	6	5.39	0	4	-5.8	2	2	0	1
cingulate ventral frontal L	Ctx p47r L	BA47 L	Cortex_Fronto_ParietalB	3480	-43	42	2	6.2	2	1	2.21	3	0	1	0
cingulate ventral frontal L	Ctx pOFC L	OFC L	Cortex_Limbic	4008	-13	12	-20	-3.74	0	3	-4.4	1	1	0	1
cingulate ventral frontal R	Ctx 10d R	BA10 R	Cortex_Default_ModeA	4520	10	66	6	-11.75	0	6	-3.79	0	4	0	3
cingulate ventral frontal R	Ctx 10pp R	BA10 R	Cortex_Limbic	3688	14	61	-15	-7.27	1	4	-5.91	1	2	0	1
cingulate ventral frontal R	Ctx 10r R	BA10 R	Cortex_Default_ModeA	2280	8	51	-8	-3.3	1	3	-3.39	0	2	0	1
cingulate ventral frontal R	Ctx 10v R	BA10 R	Cortex_Default_ModeA	4208	5	53	-15	-7.59	1	6	-5.53	0	2	0	1
cingulate ventral frontal R	Ctx 11l R	BA11 R	Cortex_Fronto_ParietalB	5240	26	47	-14	7.71	3	1	-5.43	2	1	1	0
cingulate ventral frontal R	Ctx 13l R	BA13 R	Cortex_Limbic	1784	22	29	-16	-2.69	0	2	-3.93	2	0	0	0
cingulate ventral frontal R	Ctx 47l R	BA47 R	Cortex_Default_ModeB	3344	46	32	-13	-4.03	3	2	1.51	3	2	1	0
cingulate ventral frontal R	Ctx 47m R	BA47 R	Cortex_Default_ModeB	1416	34	34	-15	4.14	2	2	3.81	2	0	0	0
cingulate ventral frontal R	Ctx 47s R	BA47 R	Cortex_Default_ModeB	4136	31	23	-20	-3.78	4	1	-4.64	2	1	1	0
cingulate ventral frontal R	Ctx OFC R	OFC R	Cortex_Limbic	7544	10	34	-22	-9.69	0	4	-7.31	0	1	0	1
cingulate ventral frontal R	Ctx a10p R	BA10 R	Cortex_Fronto_ParietalB	2600	26	60	-7	4.4	3	0	-8.25	1	3	1	0
cingulate ventral frontal R	Ctx a47r R	BA47 R	Cortex_Fronto_ParietalB	5712	39	52	-7	2.93	4	1	-10.93	1	4	1	0
cingulate ventral frontal R	Ctx p10p R	BA10 R	Cortex_Fronto_ParietalB	3760	26	59	6	-3.74	3	1	-5.02	0	3	0	0
cingulate ventral frontal R	Ctx p47r R	BA47 R	Cortex_Ventral_AttentionB	2960	45	44	-3	10.57	4	1	-2.3	3	3	1	0
cingulate ventral frontal R	Ctx pOFC R	OFC R	Cortex_Limbic	4704	14	14	-17	-5.16	0	3	2.45	1	0	0	0
cingulate vlPFC L	Ctx 44 L	BA44 L	Cortex_Default_ModeB	3440	-52	15	14	11.88	4	1	7.43	3	1	2	0
cingulate vlPFC L	Ctx 45 L	BA45 L	Cortex_Default_ModeB	4952	-50	26	3	4.06	3	1	-3.44	1	1	1	0
cingulate vlPFC L	Ctx IFJa L	IFJ L	Cortex_Fronto_ParietalA	2704	-39	13	26	2.67	2	2	6.6	3	0	2	0
cingulate vlPFC L	Ctx IFJp L	IFJ L	Cortex_Fronto_ParietalA	2088	-38	3	31	4.56	1	3	4.62	2	2	0	2
cingulate vlPFC L	Ctx IFSa L	IFS L	Cortex_Fronto_ParietalA	3376	-47	33	12	6.07	4	2	4.01	5	0	2	0
cingulate vlPFC L	Ctx IFSp L	IFS L	Cortex_Fronto_ParietalA	2160	-49	22	23	4.03	1	2	2.41	4	1	0	0
cingulate vlPFC R	Ctx 44 R	BA44 R	Cortex_Default_ModeB	4208	54	19	11	6.86	6	0	-4.54	3	2	2	0
cingulate vlPFC R	Ctx 45 R	BA45 R	Cortex_Default_ModeB	3376	51	28	2	4.76	5	0	-6.25	2	3	1	1
cingulate vlPFC R	Ctx IFJa R	IFJ R	Cortex_Fronto_ParietalA	2056	43	18	24	10.6	3	1	-3.2	1	3	1	1
cingulate vlPFC R	Ctx IFJp R	IFJ R	Cortex_Fronto_ParietalA	1592	38	7	27	6.53	2	2	-2.07	1	2	1	1
cingulate vlPFC R	Ctx IFSa R	IFS R	Cortex_Ventral_AttentionB	4080	48	38	6	8.42	5	1	-2.77	3	2	3	1

Structure	Region	Parcel	Network	Volume	MNI Coordinates			Experienced Pain (Hot-Warm)			Imagined Pain (Imagine-Warm)			Pain Conjunction	
					X	Y	Z	T	Activated (n)	Deactivated (n)	T	Activated (n)	Deactivated (n)	Activated (n)	Deactivated (n)
cingulate vlPFC R	Ctx IFSp R	IFS R	Cortex_Fronto_ParietalA	2672	49	28	19	6.07	3	1	7.26	0	3	1	0
hypothalamus L	hypothalamus anterior and tubular inferior L	hypothalamus L	subctx	624	-4	-0	-16	-1.65	0	3	-1.43	0	1	0	1
hypothalamus L	hypothalamus anterior and tubular superior L	hypothalamus L	subctx	576	-3	-1	-9	-2.61	0	5	-1.21	0	1	0	1
hypothalamus L	hypothalamus posterior L	hypothalamus L	subctx	248	-2	-8	-14	2.09	1	1	0.91	0	0	0	0
hypothalamus R	hypothalamus anterior and tubular inferior R	hypothalamus R	subctx	632	5	-0	-16	-2.01	0	3	-1.27	0	1	0	1
hypothalamus R	hypothalamus anterior and tubular superior R	hypothalamus R	subctx	536	4	-1	-9	-2.87	0	4	1.67	0	0	0	0
hypothalamus R	hypothalamus posterior R	hypothalamus R	subctx	232	3	-8	-15	-1.32	1	1	2.58	0	0	0	0
insula anterior L	Ctx AAIC L	anterior agranular insula L	Cortex_Ventral_AttentionB	1824	-33	14	-11	8.53	3	0	3.37	0	3	0	0
insula anterior L	Ctx AVI L	anterior agranular insula L	Cortex_Ventral_AttentionB	2432	-29	25	-2	6.37	5	0	4.34	2	1	1	0
insula anterior L	Ctx MI L	anterior agranular insula L	Cortex_Ventral_AttentionA	3072	-35	12	2	10.14	9	1	3.01	4	1	3	1
insula anterior L	Ctx Pir L	PIR L	Cortex_Limbic	2528	-31	4	-17	12	3	2	14.83	0	1	0	0
insula anterior R	Ctx AAIC R	anterior agranular insula R	Cortex_Ventral_AttentionA	2192	34	17	-11	4.9	6	1	-3.21	2	1	2	1
insula anterior R	Ctx AVI R	anterior agranular insula R	Cortex_Ventral_AttentionB	2752	31	26	-3	17.18	7	0	3.42	3	2	2	0
insula anterior R	Ctx MI R	anterior agranular insula R	Cortex_Ventral_AttentionA	2128	36	12	2	17.98	9	1	2.33	5	1	5	1
insula anterior R	Ctx Pir R	PIR R	Cortex_Ventral_AttentionA	3864	31	6	-18	11.46	5	2	-2.74	1	2	0	0
insula operculum L	Ctx FOP2 L	posterior operculum L	Cortex_SomatomotorB	1064	-42	-4	15	9.26	8	0	4.94	1	2	1	0
insula operculum L	Ctx FOP3 L	posterior operculum L	Cortex_Ventral_AttentionA	1296	-34	3	14	15.36	9	1	3.8	2	1	2	1
insula operculum L	Ctx FOP4 L	anterior operculum L	Cortex_Ventral_AttentionA	3752	-41	13	7	12.87	8	0	3.64	3	0	2	0
insula operculum L	Ctx FOP5 L	anterior operculum L	Cortex_Ventral_AttentionA	2376	-35	28	6	6.03	7	0	1.72	3	2	2	0
insula operculum R	Ctx FOP2 R	posterior operculum R	Cortex_SomatomotorB	1056	40	-1	16	8.74	6	0	2.38	0	2	0	0
insula operculum R	Ctx FOP3 R	posterior operculum R	Cortex_Ventral_AttentionA	872	33	8	12	10.97	9	1	2.35	4	0	4	1
insula operculum R	Ctx FOP4 R	anterior operculum R	Cortex_Ventral_AttentionA	2296	38	15	10	15.13	9	1	3.2	3	0	3	1

Structure	Region	Parcel	Network	Volume	MNI Coordinates X	Y	Z	Experienced Pain (Hot-Warm) T	Activated (n)	Deactivated (n)	Imagined Pain (Imagine-Warm) T	Activated (n)	Deactivated (n)	Pain Conjunction Activated (n)	Deactivated (n)
insula operculum R	Ctx FOP5 R	anterior operculum R	Cortex_Ventral_AttentionB	2696	37	29	6	13.97	7	0	2.53	2	3	1	0
insula posterior L	Ctx 52 L	posterior granular insula L	Cortex_SomatomotorB	1152	-38	-21	0	15.85	7	1	9.45	1	2	1	0
insula posterior L	Ctx Ig L	posterior granular insula L	Cortex_SomatomotorB	1616	-35	-14	15	6.73	8	0	5.15	1	2	1	0
insula posterior L	Ctx PI L	inferior dysgranular insula L	Cortex_Limbic	1400	-43	-4	-13	10.27	8	1	5.82	1	2	1	1
insula posterior L	Ctx PoI1 L	inferior dysgranular insula L	Cortex_Ventral_AttentionA	1960	-38	-10	-4	14.6	9	1	2.07	2	1	2	0
insula posterior L	Ctx PoI2 L	inferior dysgranular insula L	Cortex_Ventral_AttentionA	3296	-40	-1	-1	11.75	9	1	3.01	3	2	2	0
insula posterior R	Ctx 52 R	posterior granular insula R	Cortex_SomatomotorB	800	39	-19	1	6.18	7	1	4.07	0	2	0	0
insula posterior R	Ctx Ig R	posterior granular insula R	Cortex_SomatomotorB	1432	36	-11	13	12.57	8	0	4.36	1	2	0	0
insula posterior R	Ctx PI R	inferior dysgranular insula R	Cortex_Ventral_AttentionA	1528	44	-3	-12	7	8	1	-9.8	0	3	0	0
insula posterior R	Ctx PoI1 R	inferior dysgranular insula R	Cortex_Ventral_AttentionA	2048	38	-8	-6	11.18	9	1	-6.2	0	2	0	0
insula posterior R	Ctx PoI2 R	inferior dysgranular insula R	Cortex_Ventral_AttentionA	3640	40	-0	-0	13.17	9	1	-2.35	2	2	3	1
parietal TPOJ L	Ctx PSL L	PSL L	Cortex_Ventral_AttentionA	2768	-56	-44	23	2.98	2	4	-3.83	3	3	1	2
parietal TPOJ L	Ctx STV L	STV L	Cortex_Temporal_Parietal	2368	-61	-50	17	-8.01	1	4	-3.72	3	2	0	3
parietal TPOJ L	Ctx TPOJ1 L	TPOJ L	Cortex_Temporal_Parietal	2976	-52	-45	10	-5.04	0	5	-5.03	2	3	0	2
parietal TPOJ L	Ctx TPOJ2 L	TPOJ L	Cortex_Dorsal_AttentionA	3152	-52	-61	12	-7.77	1	6	-6.86	1	4	1	3
parietal TPOJ L	Ctx TPOJ3 L	TPOJ L	Cortex_Dorsal_AttentionA	1264	-42	-71	19	-6.51	0	4	-4.3	0	3	0	2
parietal TPOJ R	Ctx PSL R	PSL R	Cortex_Temporal_Parietal	3128	64	-35	26	15.18	7	0	-5.17	2	2	1	2
parietal TPOJ R	Ctx STV R	STV R	Cortex_Temporal_Parietal	3384	59	-42	19	-2.36	3	3	-4.76	2	3	2	2
parietal TPOJ R	Ctx TPOJ1 R	TPOJ R	Cortex_Temporal_Parietal	6104	53	-42	10	-6.9	2	3	-9.04	2	2	1	2
parietal TPOJ R	Ctx TPOJ2 R	TPOJ R	Cortex_Dorsal_AttentionA	3376	53	-57	8	-10.59	1	4	-10.29	1	2	1	1
parietal TPOJ R	Ctx TPOJ3 R	TPOJ R	Cortex_Default_ModeC	2696	42	-62	17	-7.02	0	4	-14	0	3	0	2
parietal inferior lobule L	Ctx IP0 L	posterior IPL L	Cortex_Dorsal_AttentionA	2192	-31	-75	27	-5.17	0	4	-4.79	0	6	0	3
parietal inferior lobule L	Ctx IP1 L	superior IPL L	Cortex_Fronto_ParietalA	3040	-31	-68	40	-3.94	0	4	5.17	1	1	0	1
parietal inferior lobule L	Ctx IP2 L	superior IPL L	Cortex_Fronto_ParietalA	3728	-39	-51	39	4.75	3	1	3.49	4	0	1	0
parietal inferior lobule L	Ctx PF L	anterior IPL L	Cortex_Ventral_AttentionA	7520	-58	-38	39	11.05	6	0	-5.25	4	1	3	0
parietal inferior lobule L	Ctx PFm L	middle IPL L	Cortex_Fronto_ParietalB	9640	-49	-56	44	-7.17	2	2	3.54	4	0	1	0
parietal inferior lobule L	Ctx PFop L	anterior IPL L	Cortex_Dorsal_AttentionB	3080	-62	-23	27	8.35	9	1	-1.96	4	2	4	0
parietal inferior lobule L	Ctx PFt L	anterior IPL L	Cortex_Dorsal_AttentionB	3664	-53	-27	38	-3.81	3	3	3.59	2	3	2	1

Structure	Region	Parcel	Network	Volume	MNI Coordinates X	Y	Z	Experienced Pain (Hot-Warm) T	Activated (n)	Deactivated (n)	Imagined Pain (Imagine-Warm) T	Activated (n)	Deactivated (n)	Pain Conjunction Activated (n)	Deactivated (n)
parietal inferior lobule L	Ctx PGi L	inferior IPL L	Cortex_Default_ModeB	7832	-45	-59	24	-22.86	0	6	3.81	2	0	0	0
parietal inferior lobule L	Ctx PGp L	posterior IPL L	Cortex_Visual_Central	3016	-37	-84	25	-7.46	0	4	-15.25	0	6	0	4
parietal inferior lobule L	Ctx PGs L	inferior IPL L	Cortex_Default_ModeA	5256	-40	-74	39	-10.14	0	4	-12.2	1	2	0	1
parietal inferior lobule R	Ctx IP0 R	posterior IPL R	Cortex_Dorsal_AttentionA	1936	34	-69	31	-6.72	0	3	-4.2	0	5	0	3
parietal inferior lobule R	Ctx IP1 R	superior IPL R	Cortex_Dorsal_AttentionA	4000	35	-63	44	7.52	1	3	-6.49	0	4	0	2
parietal inferior lobule R	Ctx IP2 R	superior IPL R	Cortex_Fronto_ParietalA	3648	43	-43	43	6.54	4	2	-5.55	0	3	0	0
parietal inferior lobule R	Ctx PF R	anterior IPL R	Cortex_Ventral_AttentionA	5704	60	-29	38	16.91	7	0	-5.65	1	3	2	1
parietal inferior lobule R	Ctx PFm R	middle IPL R	Cortex_Fronto_ParietalB	10704	53	-47	45	12.37	4	1	-6.16	1	3	0	0
parietal inferior lobule R	Ctx PFop R	anterior IPL R	Cortex_Ventral_AttentionA	2608	63	-18	26	7.85	8	0	-4.15	0	2	0	1
parietal inferior lobule R	Ctx PFt R	anterior IPL R	Cortex_Dorsal_AttentionB	2968	56	-21	40	7.6	1	5	3.1	1	2	1	1
parietal inferior lobule R	Ctx PGi R	inferior IPL R	Cortex_Default_ModeC	8568	50	-57	26	-10.99	0	5	-6.84	0	3	0	2
parietal inferior lobule R	Ctx PGp R	posterior IPL R	Cortex_Default_ModeC	4872	42	-78	27	-11.62	0	5	-7.44	0	5	0	3
parietal inferior lobule R	Ctx PGs R	inferior IPL R	Cortex_Default_ModeA	4424	45	-67	44	8.95	1	2	-5.1	0	4	0	1
parietal superior lobule L	Ctx 7AL L	anterior BA7 L	Cortex_Dorsal_AttentionB	3264	-20	-51	69	-5.58	1	4	-4.71	1	3	0	2
parietal superior lobule L	Ctx 7Am L	anterior BA7 L	Cortex_Dorsal_AttentionB	4384	-8	-59	63	-4.09	1	4	-5.04	1	2	0	2
parietal superior lobule L	Ctx 7PC L	postcentral BA7 L	Cortex_Dorsal_AttentionB	4136	-36	-48	63	-12.5	0	5	-6.6	1	3	0	2
parietal superior lobule L	Ctx 7PL L	posterior BA7 L	Cortex_Dorsal_AttentionA	1952	-14	-72	57	-3.29	0	5	2.53	2	2	0	2
parietal superior lobule L	Ctx 7Pm L	posterior BA7 L	Cortex_Fronto_ParietalC	2016	-5	-68	53	-4.94	2	2	4.31	0	3	0	2
parietal superior lobule L	Ctx AIP L	ventral intraparietal L	Cortex_Fronto_ParietalA	5032	-37	-40	44	-3.23	1	4	3.97	2	0	0	0
parietal superior lobule L	Ctx LIPd L	ventral intraparietal L	Cortex_Fronto_ParietalA	2240	-28	-55	41	2.78	0	2	3.19	4	1	0	0
parietal superior lobule L	Ctx LIPv L	dorsal intraparietal L	Cortex_Dorsal_AttentionA	2848	-30	-58	56	-8.43	0	5	-5.67	1	3	0	2
parietal superior lobule L	Ctx MIP L	MIP L	Cortex_Dorsal_AttentionA	3160	-23	-64	47	-3.84	0	4	4.31	2	2	0	1
parietal superior lobule L	Ctx VIP L	dorsal intraparietal L	Cortex_Dorsal_AttentionA	2776	-23	-64	63	-6.32	0	5	-3.98	1	3	0	3
parietal superior lobule R	Ctx 7AL R	anterior BA7 R	Cortex_Dorsal_AttentionB	3016	23	-50	69	-5.89	2	3	-5.29	0	3	0	2
parietal superior lobule R	Ctx 7Am R	anterior BA7 R	Cortex_Dorsal_AttentionB	4008	10	-58	65	-12.7	0	5	-6.17	1	2	0	2
parietal superior lobule R	Ctx 7PC R	postcentral BA7 R	Cortex_Dorsal_AttentionA	4904	35	-47	63	-13.16	1	6	-5.9	0	5	0	2
parietal superior lobule R	Ctx 7PL R	posterior BA7 R	Cortex_Fronto_ParietalC	1600	17	-69	59	-4.26	0	5	-4.07	2	2	0	1

					MNI Coordinates			Experienced Pain (Hot-Warm)			Imagined Pain (Imagine-Warm)			Pain Conjunction	
Structure	Region	Parcel	Network	Volume	X	Y	Z	T	Activated (n)	Deactivated (n)	T	Activated (n)	Deactivated (n)	Activated (n)	Deactivated (n)
parietal superior lobule R	Ctx 7Pm R	posterior BA7 R	Cortex_Fronto_ParietalC	2088	7	-67	55	-7.59	1	4	-2.85	0	3	0	2
parietal superior lobule R	Ctx AIP R	ventral intraparietal R	Cortex_Fronto_ParietalA	5640	37	-38	45	-8	1	6	2.84	1	3	0	1
parietal superior lobule R	Ctx LIPd R	ventral intraparietal R	Cortex_Dorsal_AttentionA	1128	30	-51	42	2.59	1	2	-2.32	1	2	0	1
parietal superior lobule R	Ctx LIPv R	dorsal intraparietal R	Cortex_Dorsal_AttentionA	2024	28	-55	55	-7.44	0	5	-3.65	0	4	0	3
parietal superior lobule R	Ctx MIP R	MIP R	Cortex_Dorsal_AttentionA	3672	26	-64	50	-4.48	0	5	-4.86	0	4	0	2
parietal superior lobule R	Ctx VIP R	dorsal intraparietal R	Cortex_Dorsal_AttentionA	1784	23	-61	64	-6.27	0	5	-4.4	0	3	0	2
somatomotor operculum L	Ctx 43 L	FOP1-BA43 L	Cortex_SomatomotorB	2160	-57	-0	11	7.72	9	1	4.79	6	0	6	1
somatomotor operculum L	Ctx FOP1 L	FOP1-BA43 L	Cortex_Ventral_AttentionA	1008	-50	2	5	11.32	8	0	3.03	4	1	3	0
somatomotor operculum L	Ctx OP1 L	SII+ L	Cortex_SomatomotorB	2704	-48	-21	21	8.55	7	1	-3.73	0	3	0	1
somatomotor operculum L	Ctx OP2 3 L	SII+ L	Cortex_SomatomotorB	1552	-40	-16	20	7.76	8	0	3.48	0	2	0	0
somatomotor operculum L	Ctx OP4 L	OP4 L	Cortex_SomatomotorB	2784	-59	-13	16	10.94	9	1	-5.5	3	2	3	1
somatomotor operculum L	Ctx PFcm L	SII+ L	Cortex_Ventral_AttentionA	2360	-51	-32	22	7.16	7	1	-4.49	1	3	0	2
somatomotor operculum R	Ctx 43 R	FOP1-BA43 R	Cortex_SomatomotorB	3184	56	1	11	14.88	9	1	7.65	4	1	4	0
somatomotor operculum R	Ctx FOP1 R	FOP1-BA43 R	Cortex_Ventral_AttentionA	1216	47	4	7	9.19	9	1	2.87	4	1	4	1
somatomotor operculum R	Ctx OP1 R	SII+ R	Cortex_SomatomotorB	1456	44	-19	21	11.42	7	1	-1.68	0	2	0	0
somatomotor operculum R	Ctx OP2 3 R	SII+ R	Cortex_SomatomotorB	1248	39	-12	19	12.78	9	1	2.82	1	2	1	0
somatomotor operculum R	Ctx OP4 R	OP4 R	Cortex_SomatomotorB	3664	58	-11	16	8.03	8	0	-7.64	0	2	0	0
somatomotor operculum R	Ctx PFcm R	SII+ R	Cortex_SomatomotorB	2472	49	-26	24	5.71	7	1	-2.55	0	4	0	1
somatomotor paracentral lobule L	Ctx 24dd L	cingulate motor area L	Cortex_SomatomotorA	4440	-7	-16	51	-3.24	2	3	4.72	3	1	2	1
somatomotor paracentral lobule L	Ctx 24dv L	cingulate motor area L	Cortex_Ventral_AttentionA	2112	-10	-2	46	5.59	4	0	2.83	4	1	3	0
somatomotor paracentral lobule L	Ctx 5L L	BA5 L	Cortex_SomatomotorA	2680	-13	-46	72	-3.22	2	3	2.33	0	2	0	1
somatomotor paracentral lobule L	Ctx 5m L	BA5 L	Cortex_SomatomotorA	2200	-6	-40	63	-2.94	1	4	-3.26	2	3	1	2
somatomotor paracentral lobule L	Ctx 5mv L	BA5 L	Cortex_Ventral_AttentionA	2216	-14	-36	51	-2.09	2	3	2.9	1	2	0	2
somatomotor paracentral lobule L	Ctx 6ma L	SMA L	Cortex_Fronto_ParietalA	5680	-19	4	69	-5.38	3	1	4.12	5	0	2	0
somatomotor paracentral lobule L	Ctx 6mp L	SMA L	Cortex_SomatomotorA	6432	-12	-15	71	-12.07	4	3	5.05	5	1	3	1
somatomotor paracentral lobule L	Ctx SCEF L	SMA L	Cortex_Ventral_AttentionA	5824	-6	4	59	6.01	4	0	4.57	7	0	3	0
somatomotor paracentral lobule R	Ctx 24dd R	cingulate motor area R	Cortex_SomatomotorA	4864	8	-17	53	-4.93	2	3	5.95	3	1	2	1

Structure	Region	Parcel	Network	Volume	MNI Coordinates			Experienced Pain (Hot-Warm)			Imagined Pain (Imagine-Warm)			Pain Conjunction	
					X	Y	Z	T	Activated (n)	Deactivated (n)	T	Activated (n)	Deactivated (n)	Activated (n)	Deactivated (n)
somatomotor paracentral lobule R	Ctx 24dv R	cingulate motor area R	Cortex_Ventral_AttentionA	2336	11	-4	47	5.44	4	2	8.46	3	1	2	0
somatomotor paracentral lobule R	Ctx 5L R	BA5 R	Cortex_Dorsal_AttentionB	2936	13	-45	75	-2.78	3	3	-3.93	0	2	0	1
somatomotor paracentral lobule R	Ctx 5m R	BA5 R	Cortex_SomatomotorA	2896	6	-38	66	-2.87	1	4	4.44	2	2	1	1
somatomotor paracentral lobule R	Ctx 5mv R	BA5 R	Cortex_Ventral_AttentionA	3000	14	-39	54	-7.21	1	4	4.8	2	2	1	1
somatomotor paracentral lobule R	Ctx 6ma R	SMA R	Cortex_Ventral_AttentionA	5728	20	3	68	-10.89	5	0	6.56	3	3	2	1
somatomotor paracentral lobule R	Ctx 6mp R	SMA R	Cortex_SomatomotorA	5808	17	-12	68	-6.19	1	4	5.07	4	1	1	1
somatomotor paracentral lobule R	Ctx SCEF R	SMA R	Cortex_Ventral_AttentionA	4776	7	3	59	7.16	6	1	5.56	6	0	4	0
somatomotor premotor L	Ctx 55b L	55b L	Cortex_Default_ModeB	3016	-48	-1	50	-4.62	1	3	-3.94	4	1	3	0
somatomotor premotor L	Ctx 6a L	superior premotor L	Cortex_Dorsal_AttentionB	8120	-25	-3	54	-18.74	1	3	6.48	3	1	0	1
somatomotor premotor L	Ctx 6d L	superior premotor L	Cortex_SomatomotorA	4200	-34	-14	67	-10.13	0	5	3.9	1	3	0	1
somatomotor premotor L	Ctx 6r L	inferior premotor L	Cortex_Ventral_AttentionA	4360	-52	6	19	8.77	6	0	7.1	5	0	4	0
somatomotor premotor L	Ctx 6v L	inferior premotor L	Cortex_SomatomotorB	2096	-59	4	33	-4.55	5	0	7.62	5	1	3	0
somatomotor premotor L	Ctx FEF L	superior premotor L	Cortex_Dorsal_AttentionB	2888	-39	-5	50	-4.29	2	1	4.49	6	0	2	0
somatomotor premotor L	Ctx PEF L	inferior premotor L	Cortex_Dorsal_AttentionB	1272	-48	-1	39	-2.54	1	2	3.7	2	3	1	1
somatomotor premotor R	Ctx 55b R	55b R	Cortex_Ventral_AttentionA	1880	51	3	48	-3.61	4	2	3.59	3	2	3	0
somatomotor premotor R	Ctx 6a R	superior premotor R	Cortex_Dorsal_AttentionB	7056	27	-2	52	-5.82	1	4	6.27	3	2	1	0
somatomotor premotor R	Ctx 6d R	superior premotor R	Cortex_SomatomotorA	3784	37	-12	65	-8.4	1	5	3.65	2	2	1	2
somatomotor premotor R	Ctx 6r R	inferior premotor R	Cortex_Ventral_AttentionA	5496	52	10	15	10.85	8	0	-6.95	5	0	4	0
somatomotor premotor R	Ctx 6v R	inferior premotor R	Cortex_SomatomotorB	3696	59	7	31	4.86	5	1	4.01	4	1	3	0
somatomotor premotor R	Ctx FEF R	superior premotor R	Cortex_Dorsal_AttentionB	3128	43	-1	51	-4.16	3	1	2.8	5	1	2	1
somatomotor premotor R	Ctx PEF R	inferior premotor R	Cortex_Fronto_ParietalA	1640	47	3	37	2.2	4	1	2.46	3	2	1	1
somatomotor primary L	Ctx 1 L	S1 L	Cortex_SomatomotorA	8384	-44	-26	60	-12.14	1	5	3.31	1	3	0	2
somatomotor primary L	Ctx 2 L	S1 L	Cortex_Dorsal_AttentionB	8488	-36	-34	54	-11.77	1	5	5.8	3	2	0	2
somatomotor primary L	Ctx 3a L	S1 L	Cortex_SomatomotorA	4752	-33	-21	44	-8.55	1	4	19.14	3	1	1	0
somatomotor primary L	Ctx 3b L	S1 L	Cortex_SomatomotorA	8320	-35	-24	55	-7.49	1	4	6.97	3	1	1	2
somatomotor primary L	Ctx 4 L	M1 L	Cortex_SomatomotorA	14672	-26	-19	56	-8.21	1	4	9.27	4	0	1	1
somatomotor primary R	Ctx 1 R	S1 R	Cortex_SomatomotorA	8464	46	-22	58	-17.65	1	6	-2.23	2	2	1	2

Structure	Region	Parcel	Network	Volume	MNI Coordinates			Experienced Pain (Hot-Warm)			Imagined Pain (Imagine-Warm)			Pain Conjunction	
					X	Y	Z	T	Activated (n)	Deactivated (n)	T	Activated (n)	Deactivated (n)	Activated (n)	Deactivated (n)
somatomotor primary R	Ctx 2 R	S1 R	Cortex_Dorsal_AttentionB	8240	36	-31	54	-24.84	2	6	10.09	2	1	1	1
somatomotor primary R	Ctx 3a R	S1 R	Cortex_SomatomotorA	4648	32	-20	46	-5.17	1	4	5.17	3	1	1	1
somatomotor primary R	Ctx 3b R	S1 R	Cortex_SomatomotorA	6840	36	-21	54	-11.29	1	5	4.5	3	1	1	1
somatomotor primary R	Ctx 4 R	M1 R	Cortex_SomatomotorA	13608	27	-17	58	-7.9	1	5	7.61	3	1	1	1
temporal lateral L	Ctx PHT L	middle temporal gyrus L	Cortex_Fronto_ParietalA	5224	-58	-59	0	-10.29	0	4	-3.47	2	3	0	2
temporal lateral L	Ctx TE1a L	temporal pole L	Cortex_Default_ModeB	5368	-61	-7	-20	-11.26	0	5	-4.78	0	6	0	4
temporal lateral L	Ctx TE1m L	middle temporal gyrus L	Cortex_Fronto_ParietalB	3888	-64	-26	-14	-6.58	0	4	-3.73	1	2	0	1
temporal lateral L	Ctx TE1p L	middle temporal gyrus L	Cortex_Fronto_ParietalB	8568	-61	-46	-11	-2.91	1	3	-3.98	2	1	0	0
temporal lateral L	Ctx TE2a L	inferior temporal sulcus L	Cortex_Limbic	7464	-56	-24	-27	6.77	0	2	-3.39	0	0	0	0
temporal lateral L	Ctx TE2p L	inferior temporal sulcus L	Cortex_Dorsal_AttentionA	4736	-47	-42	-19	-5.06	0	2	9.2	2	2	0	1
temporal lateral L	Ctx TF L	inferior temporal sulcus L	Cortex_Limbic	7104	-42	-24	-26	-8.13	0	5	11.78	1	1	0	0
temporal lateral L	Ctx TGd L	temporal pole L	Cortex_Default_ModeB	15600	-40	10	-32	-14.25	0	5	7.92	1	2	0	0
temporal lateral L	Ctx TGv L	temporal pole L	Cortex_Limbic	5984	-38	-2	-43	-9.4	0	3	10.78	0	0	0	0
temporal lateral R	Ctx PHT R	middle temporal gyrus R	Cortex_Dorsal_AttentionB	4912	60	-55	-3	-5.14	1	4	-6.8	1	1	1	0
temporal lateral R	Ctx TE1a R	temporal pole R	Cortex_Default_ModeA	5136	61	-2	-23	-13.38	0	5	-9.44	0	4	0	3
temporal lateral R	Ctx TE1m R	middle temporal gyrus R	Cortex_Fronto_ParietalB	4112	65	-23	-15	-9.7	0	4	-3.81	0	4	0	2
temporal lateral R	Ctx TE1p R	middle temporal gyrus R	Cortex_Fronto_ParietalB	8528	62	-42	-12	-5.59	0	2	-4.55	1	2	0	0
temporal lateral R	Ctx TE2a R	inferior temporal sulcus R	Cortex_Limbic	8192	55	-17	-29	-6.9	0	3	-4.65	0	3	0	0
temporal lateral R	Ctx TE2p R	inferior temporal sulcus R	Cortex_Dorsal_AttentionA	4552	48	-37	-19	-4.64	2	2	-5.91	3	0	0	0
temporal lateral R	Ctx TF R	inferior temporal sulcus R	Cortex_Limbic	7096	41	-22	-27	-6.28	0	5	-8.25	0	2	0	0
temporal lateral R	Ctx TGd R	temporal pole R	Cortex_Limbic	13248	38	14	-34	-10.81	0	3	-5.9	0	1	0	0
temporal lateral R	Ctx TGv R	temporal pole R	Cortex_Limbic	6256	36	-1	-45	-12.5	0	3	3.44	0	1	0	0
temporal medial L	Ctx EC L	hippocampal formation L	Cortex_Limbic	2824	-21	-12	-31	-5.04	0	5	3.66	0	1	0	1
temporal medial L	Ctx PHA1 L	PHA L	Cortex_Default_ModeC	2200	-23	-36	-15	-7.58	0	5	-3.52	0	1	0	1
temporal medial L	Ctx PHA2 L	PHA L	Cortex_Default_ModeC	688	-31	-38	-11	-7.91	0	5	-4.35	0	4	0	3
temporal medial L	Ctx PHA3 L	PHA L	Cortex_Default_ModeC	2352	-31	-38	-17	-12.16	0	5	-7.14	0	5	0	4
temporal medial L	Ctx PeEc L	PeEc L	Cortex_Limbic	8280	-31	-9	-32	-4	0	4	5.63	0	1	0	1
temporal medial L	Ctx PreS L	hippocampal formation L	Cortex_Default_ModeC	2056	-16	-32	-12	-7.67	0	4	3.84	0	2	0	0
temporal medial R	Ctx EC R	hippocampal formation R	Cortex_Default_ModeC	2272	20	-12	-30	-7.59	0	5	-5.27	0	2	0	1
temporal medial R	Ctx PHA1 R	PHA R	Cortex_Visual_Peripheral	1840	23	-34	-16	-7.86	0	6	-7.15	0	3	0	2
temporal medial R	Ctx PHA2 R	PHA R	Cortex_Default_ModeC	672	31	-34	-13	-5.41	0	6	-4.99	0	2	0	1
temporal medial R	Ctx PHA3 R	PHA R	Cortex_Default_ModeC	784	35	-36	-14	-4.96	0	6	-4.75	0	3	0	1

					MNI Coordinates			Experienced Pain (Hot-Warm)			Imagined Pain (Imagine-Warm)			Pain Conjunction	
Structure	Region	Parcel	Network	Volume	X	Y	Z	T	Activated (n)	Deactivated (n)	T	Activated (n)	Deactivated (n)	Activated (n)	Deactivated (n)
temporal medial R	Ctx PeEc R	PeEc R	Cortex_Limbic	7112	28	-7	-33	-6.78	0	5	-4.49	0	1	0	0
temporal medial R	Ctx PreS R	hippocampal formation R	Cortex_Visual_Peripheral	1664	16	-33	-10	-5.58	0	5	-9.88	0	2	0	1
visual MT+ L	Ctx FST L	vMT+ L	Cortex_Fronto_ParietalA	1792	-47	-66	3	-5.09	0	4	-10.94	0	5	0	4
visual MT+ L	Ctx LO1 L	LO L	Cortex_Visual_Central	1416	-37	-82	6	-3.31	0	3	-14.72	0	6	0	3
visual MT+ L	Ctx LO2 L	LO L	Cortex_Visual_Central	1608	-42	-85	-3	-3.73	0	4	-6.11	0	7	0	4
visual MT+ L	Ctx LO3 L	LO L	Cortex_Visual_Central	1368	-46	-79	13	-4	0	5	-11.95	0	5	0	3
visual MT+ L	Ctx MST L	dMT+ L	Cortex_Visual_Central	1272	-39	-66	7	-4.3	1	3	-11.93	2	3	1	1
visual MT+ L	Ctx MT L	dMT+ L	Cortex_Visual_Central	1120	-43	-73	10	-5.04	0	5	-8.93	0	4	0	2
visual MT+ L	Ctx PH L	PH L	Cortex_Dorsal_AttentionA	5760	-45	-65	-7	-7.7	0	4	-8.9	1	5	0	3
visual MT+ L	Ctx V4t L	vMT+ L	Cortex_Visual_Central	1176	-47	-78	1	-3.59	0	5	-6.43	0	6	0	4
visual MT+ R	Ctx FST R	vMT+ R	Cortex_Dorsal_AttentionA	1912	46	-61	-1	-4.35	0	5	-8.2	0	5	0	4
visual MT+ R	Ctx LO1 R	LO R	Cortex_Visual_Central	1320	37	-78	6	-6.58	0	4	-9.94	0	5	0	3
visual MT+ R	Ctx LO2 R	LO R	Cortex_Visual_Central	1120	44	-84	-0	-4.15	0	4	-8.49	0	5	0	4
visual MT+ R	Ctx LO3 R	LO R	Cortex_Visual_Central	1312	45	-75	12	-14.8	0	5	-13.97	0	5	0	3
visual MT+ R	Ctx MST R	dMT+ R	Cortex_Dorsal_AttentionA	1584	42	-65	5	-5.29	0	4	-8.66	0	4	0	2
visual MT+ R	Ctx MT R	dMT+ R	Cortex_Dorsal_AttentionA	1464	50	-70	8	-4.95	0	5	-7.62	0	3	0	2
visual MT+ R	Ctx PH R	PH R	Cortex_Dorsal_AttentionA	5216	47	-63	-10	-10.34	0	5	-10.52	0	6	0	3
visual MT+ R	Ctx V4t R	vMT+ R	Cortex_Dorsal_AttentionA	1152	50	-75	-1	-4.59	0	5	-5.63	0	6	0	4
visual dorsal L	Ctx IPS1 L	ISP1 L	Cortex_Dorsal_AttentionA	2424	-24	-73	36	-6.06	0	4	4.42	1	2	0	2
visual dorsal L	Ctx V3A L	supplementary V3 L	Cortex_Visual_Central	3376	-17	-89	27	-3.39	0	3	-9.69	1	4	0	3
visual dorsal L	Ctx V3B L	supplementary V3 L	Cortex_Visual_Central	1520	-26	-79	18	-3.62	0	3	-6.36	0	5	0	3
visual dorsal L	Ctx V3CD L	supplementary V3 L	Cortex_Visual_Central	1488	-35	-86	13	-4.15	0	4	-6.01	0	6	0	4
visual dorsal L	Ctx V6 L	V6 L	Cortex_Visual_Peripheral	2024	-15	-79	29	-2.68	1	3	-2.3	2	3	1	2
visual dorsal L	Ctx V6A L	V6 L	Cortex_Visual_Peripheral	1048	-21	-85	43	-3.52	0	4	-3.19	1	3	0	2
visual dorsal L	Ctx V7 L	V7 L	Cortex_Visual_Central	1184	-24	-84	31	-3.85	0	5	-3.87	0	4	0	3
visual dorsal R	Ctx IPS1 R	ISP1 R	Cortex_Dorsal_AttentionA	2544	27	-69	38	-2.75	0	3	-5.48	0	5	0	2
visual dorsal R	Ctx V3A R	supplementary V3 R	Cortex_Visual_Peripheral	3960	18	-87	32	-3.75	1	4	-10.55	1	4	0	3
visual dorsal R	Ctx V3B R	supplementary V3 R	Cortex_Dorsal_AttentionA	1696	29	-74	22	-5.61	0	2	-4.09	1	4	0	2
visual dorsal R	Ctx V3CD R	supplementary V3 R	Cortex_Visual_Central	1456	37	-81	15	-12.72	0	4	-10.22	0	6	0	4
visual dorsal R	Ctx V6 R	V6 R	Cortex_Visual_Peripheral	2416	18	-77	30	-2.23	2	3	-2.9	1	3	0	2
visual dorsal R	Ctx V6A R	V6 R	Cortex_Visual_Peripheral	1032	24	-82	46	-4.03	0	3	-2.7	0	4	0	2
visual dorsal R	Ctx V7 R	V7 R	Cortex_Visual_Peripheral	1320	29	-82	34	-4.11	0	4	-3.9	0	4	0	3
visual early L	Ctx V1 L	striate L	Cortex_Visual_Peripheral	20216	-10	-85	2	4.91	3	1	-7.25	1	5	1	1
visual early L	Ctx V2 L	extrastriate L	Cortex_Visual_Peripheral	15816	-9	-79	4	-7.8	1	3	-7.62	1	5	0	3
visual early L	Ctx V3 L	extrastriate L	Cortex_Visual_Central	11552	-15	-85	8	-3.69	1	3	-6.67	1	5	1	2
visual early L	Ctx V4 L	extrastriate L	Cortex_Visual_Central	9128	-27	-86	-0	-3.53	0	4	-14.21	0	6	0	3
visual early R	Ctx V1 R	striate R	Cortex_Visual_Peripheral	21760	13	-81	4	5.81	2	1	-12.41	1	5	1	0
visual early R	Ctx V2 R	extrastriate R	Cortex_Visual_Central	15488	12	-75	5	-5.71	1	3	-3.51	1	5	0	2
visual early R	Ctx V3 R	extrastriate R	Cortex_Visual_Central	12256	19	-85	9	-4.38	1	3	-4.77	1	5	0	2
visual early R	Ctx V4 R	extrastriate R	Cortex_Visual_Central	8088	29	-83	-0	-4.26	0	3	-12.95	0	6	0	2
visual ventral L	Ctx FFC L	FFC L	Cortex_Dorsal_AttentionA	5616	-42	-61	-19	-7.09	0	4	-6.17	1	5	0	4

Structure	Region	Parcel	Network	Volume	MNI Coordinates X	Y	Z	Experienced Pain (Hot-Warm) T	Activated (n)	Deactivated (n)	Imagined Pain (Imagine-Warm) T	Activated (n)	Deactivated (n)	Pain Conjunction Activated (n)	Deactivated (n)
visual ventral L	Ctx PIT L	FFC L	Cortex_Visual_Central	2096	-38	-84	-15	-4.15	1	3	4.9	2	4	0	1
visual ventral L	Ctx V8 L	VVC L	Cortex_Visual_Central	1904	-31	-75	-12	-3.13	0	2	-5	1	6	0	1
visual ventral L	Ctx VMV1 L	VMV L	Cortex_Visual_Peripheral	1792	-19	-55	-6	-4.12	1	3	-2.1	1	3	0	3
visual ventral L	Ctx VMV2 L	VMV L	Cortex_Visual_Peripheral	1664	-29	-55	-4	-6.92	1	2	10.71	1	3	0	2
visual ventral L	Ctx VMV3 L	VMV L	Cortex_Visual_Peripheral	1312	-28	-61	-11	2.65	1	2	-4.3	1	6	0	2
visual ventral L	Ctx VVC L	VVC L	Cortex_Visual_Central	3032	-31	-52	-16	-5.85	0	4	-3.85	2	5	0	4
visual ventral R	Ctx FFC R	FFC R	Cortex_Visual_Central	6080	41	-54	-20	-4.72	0	5	-8.12	2	3	0	4
visual ventral R	Ctx PIT R	FFC R	Cortex_Visual_Central	3136	41	-80	-13	-5.88	0	3	-6.04	3	3	0	3
visual ventral R	Ctx V8 R	VVC R	Cortex_Visual_Central	2448	31	-72	-12	-3.16	1	3	-6.16	1	5	0	3
visual ventral R	Ctx VMV1 R	VMV R	Cortex_Visual_Peripheral	2320	19	-55	-7	-4.36	1	2	-6.46	1	3	0	1
visual ventral R	Ctx VMV2 R	VMV R	Cortex_Visual_Peripheral	1808	29	-51	-4	-5.57	1	2	4.63	1	3	0	1
visual ventral R	Ctx VMV3 R	VMV R	Cortex_Visual_Peripheral	1864	29	-59	-8	-6.99	1	3	-3.35	1	4	0	3
visual ventral R	Ctx VVC R	VVC R	Cortex_Visual_Central	3792	29	-45	-18	-7.49	0	6	-4.97	1	5	0	3

Notes. Voxelwise group-level robust-regression contrasts for experienced pain (Hot–Warm) and imagined pain (Imagine–Warm), summarized by CANLab-2024 atlas parcel (N = 9 participants; FDR q < .05). For each parcel we list its Structure, Region, Parcel, and Network, its volume (mm³), and its MNI centroid (X, Y, Z). Within each contrast, T is the peak (maximum-magnitude) group-level voxelwise *t* within the parcel. Activated (n) and Deactivated (n) give the number of participants (of 9) with a significant positive or negative effect within the parcel, computed from each participant's FDR-thresholded voxelwise map: a participant is counted as *activated* when the parcel's significant voxels have a positive peak or a positive mean, and as *deactivated* when the parcel's significant voxels have both a negative peak and a negative mean. Pain Conjunction reports the number of participants with a significant effect of the same sign within the parcel for both contrasts (Activated = positive in both Hot–Warm and Imagine–Warm; Deactivated = negative in both). Parcels are grouped by anatomical structure. n: Number of subjects exhibiting a significant effect. † $p < .10$, * $p < 0.05$, ** $p < 0.01$ and *** $p < .001$.

Supplementary Table S3: Pattern expression of participant-specific nociceptive body site selective SVM weight maps in a priori pain regions

	MNI Coordinates			Hot-Rest (Target Body Site)		Hot-Warm (Target Body Site)		Hot-Warm x Target – Other Body Site		Imagine-Rest (Target Body Site)		Imagine-Warm (Target Body Site)		Imagine-Warm x Target – Other Body Site	
Region	X	Y	Z	T	n	T	n	T	n	T	n	T	n	T	n
Amygdala L	-22.5	-4.5	-20.5	3.75**	7	3.65**	7	1.96†	5	-1.87†	0	1.27	3	0.410	1
Amygdala R	23.5	-2.5	-20.5												
aMCC MPFC	1.5	15.5	33.5	4.67**	8	4.36**	7	1.27	2	-1.83	1	0.94	2	-1.51	0
aIns L	-40.5	23.5	3.5	7.45***	9	7.28***	9	0.79	3	-2.10+	0	1.59	3	-0.89	1
aIns R	39.5	25.5	3.5												
mIns L	-38.5	3.5	3.5	9.27***	9	9.38***	9	0.26	2	-3.91**	0	0.19	1	-1.31	1
mIns R	37.5	3.5	3.5												
dpIns L	-40.5	-22.5	19.5	10.74***	9	1.00***	9	5.36***	8	-6.80***	0	-0.11	2	-0.55	0
dpIns R	39.5	-18.5	17.5												
S1 L	-34.5	-28.5	55.5	6.76***	9	6.81***	9	4.44**	8	-2.63*	0	1.01	4	-0.41	2
S1 R	35.5	-24.5	55.5												
S2 L	55.5	-16.5	19.5	10.53***	9	10.22***	9	2.63**	6	-4.46**	0	-0.72	1	-1.40	0
S2 R	-56.5	-22.5	19.5												
Hypothalamus	-0.5	-2.5	-12.5	2.08+	3	2.12†	4	0.81	2	-0.43	0	0.66	1	0.91	2
Thal VPLM L	17.5	-18.5	5.5	2.75*	5	2.75*	5	1.05	3	-1.67	0	1.23	2	-0.72	0
Thal VPLM R	-18.5	-20.5	5.5												
Thal IL	-0.5	-12.5	-0.5	4.33**	8	4.66**	8	2.12+	4	-2.08*	0	1.46	3	-0.76	0
Thal MD	-0.5	-14.5	5.5	3.92**	7	3.84**	7	1.49	2	-2.77**	0	0.94	2	-2.10*	0
PAG	-0.5	-32.5	-10.5	2.14*	4	2.43*	6	0.82	3	0.06	0	1.29	0	0.73	1
Parabrachial nuclei L	-6.5	-36.5	-22.5	1.85+	3	2.10†	3	2.41*	7	-1.00	0	0.51	2	0.50	3
Parabrachial nuclei R	7.5	-38.5	-22.5												
RVM	3.5	-34.5	-42.5	0.10	1	-0.06	1	-2.05†	0	-0.16	2	-0.25	1	-0.70	0

Notes. n: Number of subjects with significant pattern expression. aMCC MPFC: anterior midcingulate cortex/medial prefrontal cortex, aIns: anterior insula, mIns: medial insula, dpIns: dorsoposterior insula, S1: primary somatosensory cortex, S2: secondary somatosensory cortex, Thal: thalamus, VPLM: medial ventral posterolateral, IL: intralaminar, MD: mediodorsal, PAG: periaqueductal gray, RVM: rostral ventral medulla. † $p < .10$, * $p < 0.05$, ** $p < 0.01$ and *** $p < .001$.

Supplementary Table S4: Pattern expression of participant-specific nociceptive body site selective SVM weight maps in 131 CANlab 2024 atlas parcels

Parcel	Region	Pain Region	MNI Coordinates X	Y	Z	Hot-Rest (Target Body Site) T	n	Hot-Warm (Target Body Site) T	n	Hot-Warm x Target – Other Body Site T	n	Imagine-Rest (Target Body Site) T	n	Imagine-Warm (Target Body Site) T	n	Imagine-Warm x Target – Other Body Site T	n
55b L	somatomotor premotor L		-48.5	-0.5	49.5	4.51**	8	4.69**	8	2.18	4	-0.46	2	1.96	4	-1.12	0
55b R	somatomotor premotor R		51.5	3.5	47.5												
aCAU L	CAU L		-12.5	15.5	5.5	3.17*	7	3.13*	7	2.19	3	-0.46	0	1.43	1	0.21	0
aCAU R	CAU R		13.5	17.5	5.5												
AMY BL CeM L	Amygdala L	Amygdala	-20.5	-4.5	-18.5	3.85*	7	3.84*	7	2.37	5	-1.7	0	1.65	2	0.86	0
AMY BL CeM R	Amygdala R	Amygdala	21.5	-4.5	-18.5												
AMY La L	Amygdala L	Amygdala	-28.5	-2.5	-22.5	2.14†	4	2.16†	5	1.5	0	-1.19	0	-0.01	0	-0.62	0
AMY La R	Amygdala R	Amygdala	27.5	-2.5	-24.5												
anterior AAC L	auditory association cortex L		-52.5	-8.5	-12.5	2.98*	4	2.95*	4	1.43	2	-0.14	1	2.54	3	0.39	0
anterior AAC R	auditory association cortex R		53.5	-4.5	-14.5												
anterior agranular insula L	insula anterior L	aIns/mIns	-32.5	17.5	-2.5	7.9***	9	7.96***	9	0.28	1	-2.78	0	0.65	2	-1.06	0
anterior agranular insula R	insula anterior R	aIns/mIns	33.5	19.5	-4.5												
anterior BA7 L	parietal superior lobule L		-12.5	-54.5	65.5	5.16**	9	5.11**	9	4.45*	8	-3.6†	0	0.72	0	0.6	0
anterior BA7 R	parietal superior lobule R		15.5	-54.5	67.5												
anterior IPL L	parietal inferior lobule L		-58.5	-32.5	35.5	7.94***	9	8.39***	9	1.37	4	-3.13	0	0.33	2	-1.12	0
anterior IPL R	parietal inferior lobule R		59.5	-24.5	35.5												
anterior operculum L	insula operculum L	aIns	-38.5	19.5	7.5	9.45***	9	9.51***	9	-0.05	1	-2.38	0	1.3	2	-1.31	0
anterior operculum R	insula operculum R	aIns	37.5	21.5	7.5												
anterior precuneus L	cingulate posterior L		-6.5	-30.5	41.5	3.73*	6	3.71*	6	1.28	2	-3.97*	0	0.22	2	-1.44	0
anterior precuneus R	cingulate posterior R		9.5	-32.5	41.5												
aPUT L	PUT L		-22.5	9.5	-4.5	3.35*	6	3.2*	5	1.78	3	-0.61	1	1.13	1	0.12	1
aPUT R	PUT R		23.5	9.5	-4.5												
auditory transition area L	auditory association cortex L		-58.5	-8.5	-0.5	9.07***	9	8.9***	9	0.99	1	-1.86	1	1.58	3	-1.03	0
auditory transition area R	auditory association cortex R		59.5	-4.5	-2.5												
auditory+ L	auditory early L	dpIns	-44.5	-28.5	9.5	9.64***	9	9.4***	9	3.07†	6	-5.65*	0	-0.22	1	-0.63	0
auditory+ R	auditory early R	dpIns	47.5	-22.5	11.5												
BA10 L	cingulate ventral frontal L		-14.5	57.5	-2.5	3.48*	7	3.4*	7	0.78	2	-0.94	1	1.78	2	0.06	0

Parcel	Region	Pain Region	MNI Coordinates X	Y	Z	Hot-Rest (Target Body Site) T	n	Hot-Warm (Target Body Site) T	n	Hot-Warm x Target – Other Body Site T	n	Imagine-Rest (Target Body Site) T	n	Imagine-Warm (Target Body Site) T	n	Imagine-Warm x Target – Other Body Site T	n
BA10 R	cingulate ventral frontal R		13.5	59.5	-4.5												
BA11 L	cingulate ventral frontal L		-26.5	47.5	-12.5	5.48**	9	5.83**	9	2.45	5	-1.14	0	1.98	1	0.41	0
BA11 R	cingulate ventral frontal R		25.5	47.5	-14.5												
BA13 L	cingulate ventral frontal L		-22.5	29.5	-18.5	4.28**	8	4.48**	8	2.08	3	-2.25	0	0.35	1	-0.03	0
BA13 R	cingulate ventral frontal R		21.5	29.5	-16.5												
BA44 L	cingulate vlPFC L		-52.5	15.5	13.5	5.84**	9	6.08**	9	0.15	3	-1.05	0	1.96	4	-0.99	0
BA44 R	cingulate vlPFC R		53.5	19.5	11.5												
BA45 L	cingulate vlPFC L	aIns	-50.5	25.5	3.5	5.13**	8	5.1**	8	0.8	2	-1.41	0	1.44	3	-0.78	0
BA45 R	cingulate vlPFC R	aIns	51.5	27.5	1.5												
BA46 L	cingulate dlPFC L		-36.5	39.5	23.5	4.43**	7	4.7**	7	0.79	2	-1.47	0	1.54	4	-0.73	0
BA46 R	cingulate dlPFC R		37.5	41.5	25.5												
BA47 L	cingulate ventral frontal L		-40.5	35.5	-10.5	4.16*	7	4.35**	8	1.85	4	-2.22	0	1.98	3	-0.36	0
BA47 R	cingulate ventral frontal R		39.5	37.5	-10.5												
BA5 L	somatomotor paracentral lobule L	S1	-12.5	-40.5	63.5	6.39**	9	6.6**	9	6.32***	9	-4.23*	0	0.09	1	0.45	0
BA5 R	somatomotor paracentral lobule R	S1	11.5	-40.5	65.5												
BA9 L	cingulate dlPFC L		-20.5	49.5	33.5	3.33*	6	3.41*	6	1.83	4	-0.02	1	1.86	4	0.22	1
BA9 R	cingulate dlPFC R		19.5	55.5	29.5												
Cblm CrusI CrusII VIIIb L	Cblm cortex L		-30.5	-70.5	-38.5	3.79*	5	3.57*	5	1.16	1	-0.67	0	2.18	3	-0.23	0
Cblm CrusI CrusII VIIIb R	Cblm cortex R		31.5	-70.5	-38.5												
Cblm I IV V VI L	Cblm cortex L		-18.5	-54.5	-22.5	3.84*	5	3.75*	5	3.47†	5	-0.58	2	2.5	4	-0.72	0
Cblm I IV V VI R	Cblm cortex R		19.5	-54.5	-22.5												
Cblm vermis	Cblm vermis		-0.5	-66.5	-30.5	2.73*	5	2.8*	5	1.09	0	-1.49	0	1.68	2	-0.48	0
Cblm VIIIa VIIb IX X L	Cblm cortex L		-18.5	-54.5	-50.5	5.15**	8	5.2**	8	2.15	4	-1.75	0	2.19	4	0.45	0
Cblm VIIIa VIIb IX X R	Cblm cortex R		17.5	-54.5	-52.5												
Cholinergic nuclei L	Pons L		-2.5	-38.5	-28.5	1.54	2	1.23	2	0.55	0	0.11	0	0.36	1	0.26	0
Cholinergic nuclei R	Pons R		3.5	-36.5	-26.5												
cingulate motor area L	somatomotor paracentral lobule L		-8.5	-12.5	49.5	6.69***	9	6.17**	9	3.57†	5	-2.9	0	0.63	2	-0.66	0
cingulate motor area R	somatomotor paracentral lobule R		9.5	-12.5	51.5												
Cranial nuclei L	Medulla L		-6.5	-42.5	-42.5	1.58	2	1.6	3	0.64	0	-0.59	0	0.36	0	-0.36	0
Cranial nuclei R	Medulla R		5.5	-42.5	-44.5												

Parcel	Region	Pain Region	MNI Coordinates			Hot-Rest (Target Body Site)		Hot-Warm (Target Body Site)		Hot-Warm x Target – Other Body Site		Imagine-Rest (Target Body Site)		Imagine-Warm (Target Body Site)		Imagine-Warm x Target – Other Body Site	
			X	Y	Z	T	n	T	n	T	n	T	n	T	n	T	n
dmPFC L	cingulate ACC mPFC L		-6.5	43.5	31.5	2.81*	6	2.74*	6	1.03	3	-1.69	0	0.92	3	-0.19	0
dmPFC R	cingulate ACC mPFC R		7.5	43.5	31.5												
dMT+ L	visual MT+ L		-40.5	-68.5	7.5	3.06*	5	3.04*	5	1.13	2	-0.79	0	1.92	2	0.25	0
dMT+ R	visual MT+ R		45.5	-66.5	7.5												
dorsal intraparietal L	parietal superior lobule L		-26.5	-60.5	59.5	5.14**	9	5.12**	9	4.16**	9	-2.1	0	1.48	2	0.59	0
dorsal intraparietal R	parietal superior lobule R		25.5	-58.5	59.5												
early auditory ctx a1 L	auditory early L		-42.5	-24.5	11.5	9.08***	9	8.51***	9	3.83*	8	-4.86*	0	-0.13	1	1.28	0
early auditory ctx a1 R	auditory early R		43.5	-20.5	11.5												
extrastriate L	visual early L		-14.5	-80.5	1.5	3.16*	5	3.23*	5	1.74	2	-0.57	1	1.98	3	-0.36	0
extrastriate R	visual early R		17.5	-80.5	1.5												
FFC L	visual ventral L		-40.5	-68.5	-18.5	3.24*	6	2.98*	5	1.25	2	-0.9	0	1.82	2	-0.41	0
FFC R	visual ventral R		41.5	-62.5	-18.5												
FOP1-BA43 L	somatomotor operculum L		-54.5	1.5	9.5	10.46***	9	10.43***	9	1.28	3	-2.76	0	0.64	2	-1.13	0
FOP1-BA43 R	somatomotor operculum R		53.5	1.5	9.5												
GPe L	GP L		-20.5	-2.5	-2.5	0.86	3	0.61	3	-1.4	0	-0.48	0	-0.15	0	-0.98	0
GPe R	GP R		19.5	-0.5	-0.5												
GPi L	GP L		-18.5	-6.5	-4.5	0.28	2	0.19	2	-1.05	0	0.11	0	0.09	1	-0.34	0
GPi R	GP R		19.5	-4.5	-4.5												
Hipp L	Hippocampal Formation L		-26.5	-22.5	-14.5	2.75*	3	2.74*	4	2.11	3	-2.33	0	0.48	0	-0.02	0
Hipp R	Hippocampal Formation R		27.5	-20.5	-14.5												
hippocampal formation L	temporal medial L		-18.5	-20.5	-22.5	4.09**	7	4.63**	8	2.01	4	-1.8	0	1.65	0	-0.18	0
hippocampal formation R	temporal medial R		19.5	-20.5	-22.5												
hypothalamus L	hypothalamus L	hypothalamus	-2.5	-2.5	-12.5	2.08†	3	2.12†	4	0.81	1	-0.43	0	0.66	0	0.91	0
hypothalamus R	hypothalamus R	hypothalamus	3.5	-2.5	-12.5												
IFJ L	cingulate vlPFC L		-38.5	7.5	27.5	3.59*	6	3.75*	6	1.19	2	-0.94	0	1.45	4	-1.01	0
IFJ R	cingulate vlPFC R		41.5	13.5	25.5												
IFS L	cingulate vlPFC L		-48.5	27.5	15.5	4.37**	8	4.38**	7	0.39	2	-1.69	0	1.41	4	-1.34	0
IFS R	cingulate vlPFC R		47.5	33.5	11.5												
inferior dysgranular insula L	insula posterior L	mIns	-40.5	-4.5	-4.5	6.93***	9	6.91***	9	0.41	1	-4.56*	0	-0.84	1	-1.62	0
inferior dysgranular insula R	insula posterior R	mIns	41.5	-2.5	-4.5												

Parcel	Region	Pain Region	MNI Coordinates X	Y	Z	Hot-Rest (Target Body Site) T	n	Hot-Warm (Target Body Site) T	n	Hot-Warm x Target – Other Body Site T	n	Imagine-Rest (Target Body Site) T	n	Imagine-Warm (Target Body Site) T	n	Imagine-Warm x Target – Other Body Site T	n
inferior IPL L	parietal inferior lobule L		-42.5	-64.5	29.5	2.41†	5	2.59*	5	1.25	2	-2.02	0	0.94	2	-0.42	0
inferior IPL R	parietal inferior lobule R		47.5	-60.5	31.5												
Inferior lateral BA8 L	cingulate dlPFC L		-38.5	13.5	47.5	3.15*	5	3.3*	5	2.61	5	-2.58	0	0.86	2	-0.91	0
Inferior lateral BA8 R	cingulate dlPFC R		39.5	15.5	45.5												
inferior premotor L	somatomotor premotor L		-52.5	3.5	25.5	6.74***	9	7.08***	9	0.58	2	-0.85	0	1.88	4	-1.97	0
inferior premotor R	somatomotor premotor R		53.5	7.5	23.5												
inferior temporal sulcus L	temporal lateral L		-48.5	-28.5	-24.5	2.56*	5	2.41†	4	1.22	2	-1.31	0	1.45	2	-0.58	0
inferior temporal sulcus R	temporal lateral R		49.5	-22.5	-26.5												
infralimbic L	cingulate ACC mPFC L		-4.5	23.5	-10.5	1.73	3	1.57	3	0.04	1	-0.89	0	0.21	0	-0.5	0
infralimbic R	cingulate ACC mPFC R		3.5	23.5	-10.5												
ISP1 L	visual dorsal L		-24.5	-72.5	35.5	2.8*	5	2.92*	5	1.5	2	-1.45	1	0.6	3	-0.08	0
ISP1 R	visual dorsal R		27.5	-68.5	37.5												
PnO PnC L	Pons L	RVM	-2.5	-34.5	-32.5	-0.11	0	-0.36	0	-0.97	0	-0.25	0	-0.48	0	-0.51	0
PnO PnC R	Pons R	RVM	3.5	-34.5	-32.5												
LC+ L	Pons L		-6.5	-36.5	-34.5	0.39	2	0.16	1	-0.13	1	-0.67	0	-0.46	0	-0.31	0
LC+ R	Pons R	RVM	7.5	-36.5	-34.5												
LO L	visual MT+ L		-40.5	-82.5	5.5	2.62*	5	2.61*	5	2.33	4	0.06	1	1.92	3	0.25	0
LO R	visual MT+ R		41.5	-78.5	5.5												
M1 L	somatomotor primary L		-26.5	-20.5	55.5	6.39**	9	6.51**	9	4.91*	9	-1.92	0	1.26	3	0.33	0
M1 R	somatomotor primary R		25.5	-18.5	57.5												
Medullary Raphe (Serotonergic)	Medulla	RVM	-0.5	-36.5	-52.5	-0.31	0	-0.17	1	-0.52	0	1.02	0	0.68	1	0.31	0
Medullary reticular formation L	Medulla L		-6.5	-42.5	-54.5	1.8	4	2.23†	6	0.57	0	-0.53	0	0.66	0	0.67	0
Medullary reticular formation R	Medulla R	RVM	5.5	-42.5	-54.5												
midcingulate L	cingulate ACC mPFC L	aMCC MPFC	-6.5	19.5	29.5	4.48**	7	4.23**	7	1.63	2	-2.12	1	0.94	2	-1.14	0
midcingulate R	cingulate ACC mPFC R	aMCC MPFC	7.5	17.5	31.5												
middle IPL L	parietal inferior lobule L		-48.5	-56.5	43.5	3.63*	5	3.94*	5	1.82	1	-2.55	0	0.66	3	-1.02	0
middle IPL R	parietal inferior lobule R		53.5	-46.5	45.5												

Parcel	Region	Pain Region	MNI Coordinates X	Y	Z	Hot-Rest (Target Body Site) T	n	Hot-Warm (Target Body Site) T	n	Hot-Warm x Target – Other Body Site T	n	Imagine-Rest (Target Body Site) T	n	Imagine-Warm (Target Body Site) T	n	Imagine-Warm x Target – Other Body Site T	n
middle temporal gyrus L	temporal lateral L		-60.5	-44.5	-8.5	3.92*	6	3.81*	6	1.76	3	-2.76	0	0.6	1	-0.09	0
middle temporal gyrus R	temporal lateral R		61.5	-40.5	-10.5												
MIP L	parietal superior lobule L		-22.5	-64.5	47.5	3.83*	7	4.44**	7	2.65	4	-0.89	0	2.34	3	1.86	0
MIP R	parietal superior lobule R		25.5	-64.5	49.5												
OFC L	cingulate ventral frontal L		-12.5	23.5	-20.5	3.68*	6	3.84*	6	2.44	4	-2.42	0	1.3	3	0.53	0
OFC R	cingulate ventral frontal R		11.5	25.5	-20.5												
Olivary complex L	Medulla L		-6.5	-36.5	-52.5	1.34	2	1.42	2	0.44	2	-1.63	0	0.11	1	0.27	0
Olivary complex R	Medulla R	RVM	5.5	-36.5	-52.5												
OP4 L	somatomotor operculum L	S2	-58.5	-12.5	15.5	10.77***	9	11.57***	9	1.97	5	-3.94†	0	-0.93	1	-1.19	0
OP4 R	somatomotor operculum R	S2	57.5	-10.5	15.5												
PAG	Midbrain	PAG	-0.5	-32.5	-10.5	2.14†	4	2.43*	5	0.82	2	0.06	0	1.29	0	0.73	0
Parabrachial nuclei L	Pons L	Parabrachial nuclei	-6.5	-36.5	-22.5	1.67	3	1.85	3	1.83	2	-0.57	0	0.75	2	0.38	0
Parabrachial nuclei R	Pons R	Parabrachial nuclei	7.5	-38.5	-22.5												
parietal occipital sulcus L	cingulate posterior L		-14.5	-64.5	25.5	3.71*	5	3.79*	5	1.28	3	-1.57	0	1.54	2	-0.6	0
parietal occipital sulcus R	cingulate posterior R		17.5	-62.5	27.5												
pCAU L	CAU L		-14.5	-2.5	19.5	2.63*	4	2.64*	4	1.45	1	-0.82	0	1.31	1	-0.5	0
pCAU R	CAU R		13.5	-0.5	19.5												
PCV L	cingulate posterior L		-6.5	-50.5	51.5	4.08**	7	4.42**	8	2.21	3	-1.32	0	1.49	3	0.12	0
PCV R	cingulate posterior R		7.5	-52.5	51.5												
PeEc L	temporal medial L		-30.5	-8.5	-32.5	4.02**	8	4**	7	1.33	3	-1.42	0	0.83	1	0.45	0
PeEc R	temporal medial R		27.5	-6.5	-32.5												
PH RPH L	visual MT+ L		-44.5	-64.5	-6.5	3.3*	5	3.03*	5	2.1	5	-1.66	0	1.13	2	0.39	0
PH RPH R	visual MT+ R		47.5	-62.5	-10.5												
PHA L	temporal medial L		-28.5	-36.5	-14.5	3.46*	5	3.45*	5	0.91	1	-0.03	0	2.29	4	-0.32	0
PHA R	temporal medial R		27.5	-34.5	-14.5												
PIR L	insula anterior L		-30.5	3.5	-16.5	7.04***	9	7.64***	9	1.74	3	-3.34†	0	-0.34	1	-1.39	0
PIR R	insula anterior R		31.5	5.5	-18.5												
postcentral BA7 L	parietal superior lobule L		-36.5	-48.5	63.5	6.91***	9	6.91***	9	3.93*	8	-2.93	0	1.29	2	-0.43	0
postcentral BA7 R	parietal superior lobule R		35.5	-46.5	63.5												

Parcel	Region	Pain Region	MNI Coordinates X	Y	Z	Hot-Rest (Target Body Site) T	n	Hot-Warm (Target Body Site) T	n	Hot-Warm x Target – Other Body Site T	n	Imagine-Rest (Target Body Site) T	n	Imagine-Warm (Target Body Site) T	n	Imagine-Warm x Target – Other Body Site T	n
posterior AAC L	auditory association cortex L		-52.5	-32.5	-2.5	3.09*	5	3.24*	6	1.63	2	-1.61	0	2.21	3	0.38	0
posterior AAC R	auditory association cortex R		53.5	-28.5	-2.5												
posterior BA7 L	parietal superior lobule L		-8.5	-70.5	55.5	4.23**	7	4.27**	6	2.72	4	-1.41	0	1.88	3	0.5	0
posterior BA7 R	parietal superior lobule R		11.5	-68.5	57.5												
posterior granular insula L	insula posterior L	dpIns	-36.5	-16.5	9.5	11.07***	9	10.95***	9	4.48*	8	-6.88*	0	-0.76	0	0.37	0
posterior granular insula R	insula posterior R	dpIns	37.5	-14.5	9.5												
posterior IPL L	parietal inferior lobule L		-34.5	-80.5	25.5	3.37*	5	3.1*	5	2.04	3	-1.37	0	0.86	2	-0.08	0
posterior IPL R	parietal inferior lobule R		39.5	-76.5	27.5												
posterior operculum L	insula operculum L	mIns	-38.5	-0.5	13.5	11.85***	9	11.53***	9	1.44	2	-3.51†	0	0.56	1	-0.19	0
posterior operculum R	insula operculum R	mIns	37.5	3.5	15.5												
pPUT L	PUT L		-28.5	-6.5	3.5	3.22*	6	3.06*	6	0.71	2	-1.37	1	0.5	1	-0.98	0
pPUT R	PUT R		29.5	-6.5	1.5												
precuneus proper L	cingulate posterior L		-6.5	-52.5	31.5	2.58*	5	2.69*	5	-0.08	1	-1.85	0	0.62	1	-0.84	0
precuneus proper R	cingulate posterior R		7.5	-52.5	33.5												
PSL L	parietal TPOJ L		-56.5	-44.5	23.5	6.73***	8	6.26**	8	1.58	2	-3.35†	0	1.08	3	-0.4	0
PSL R	parietal TPOJ R		63.5	-34.5	25.5												
RN L	Midbrain L		-4.5	-18.5	-8.5	0.47	0	0.1	0	0.37	0	-0.88	0	-0.76	0	-0.79	0
RN R	Midbrain R		5.5	-18.5	-8.5												
Rostral Raphe (Serotonergic)	Midbrain		-0.5	-28.5	-12.5	1.23	1	1.39	2	0.91	0	0.23	0	0.74	0	0.58	0
Rostral reticular formation L	Midbrain L		-8.5	-26.5	-4.5	2.44*	5	2.55*	4	1.39	3	-1.03	0	0.55	1	0.45	0
Rostral reticular formation R	Midbrain R		7.5	-24.5	-4.5												
RSC L	cingulate posterior L		-4.5	-42.5	19.5	3.23*	6	3.32*	6	0.92	3	-1.85	0	1.23	1	-0.99	0
RSC R	cingulate posterior R		5.5	-40.5	21.5												
S1 L	somatomotor primary L	S1	-38.5	-26.5	53.5	6.72***	9	6.78**	9	4.21*	7	-2.45	0	1.03	2	-0.47	0
S1 R	somatomotor primary R	S1	37.5	-24.5	53.5												
Shen Med L	Medulla L		-2.5	-38.5	-60.5	2.54*	5	2.91*	6	0.56	0	-0.54	1	0.76	1	0.22	0
Shen Med R	Medulla R	RVM	7.5	-40.5	-60.5												

Parcel	Region	Pain Region	MNI Coordinates			Hot-Rest (Target Body Site)		Hot-Warm (Target Body Site)		Hot-Warm x Target – Other Body Site		Imagine-Rest (Target Body Site)		Imagine-Warm (Target Body Site)		Imagine-Warm x Target – Other Body Site	
			X	Y	Z	T	n	T	n	T	n	T	n	T	n	T	n
Shen Midb Lc	Midbrain L		-8.5	-18.5	-18.5	3.98**	7	4.57**	9	3.02†	4	-2.18	0	0.73	0	0.16	0
Shen Midb Lrd	Midbrain L		-12.5	-20.5	-6.5	2.64*	5	3.12*	6	0.73	1	-2.3	0	-0.29	0	-0.7	0
Shen Midb Rcd	Midbrain R		11.5	-20.5	-16.5	2.69*	5	2.8*	5	2.51	4	-1.34	0	1.3	2	1.26	0
Shen Midb Rrd	Midbrain R		13.5	-20.5	-6.5	3.22*	6	3.48*	6	2.93	5	-2.66	0	-0.26	0	0.15	0
Shen Pons Lcd	Pons L	RVM	-8.5	-32.5	-40.5	1.82	4	2.38†	7	0.87	2	-0.98	0	0.7	1	0.45	0
Shen Pons Lrd	Pons L		-8.5	-36.5	-28.5	3*	7	3.03*	7	2.21	4	-1.19	0	1.05	1	-0.84	0
Shen Pons Lv	Pons L		-6.5	-20.5	-34.5	2†	4	2.51*	7	1.56	1	-2.06	0	-0.35	0	-0.82	0
Shen Pons Rcd	Pons R	RVM	9.5	-34.5	-36.5	1.02	2	0.62	2	-1.16	0	-1.43	0	-0.81	1	-1.28	0
Shen Pons Rcv	Pons R	RVM	5.5	-24.5	-42.5	1.47	3	1.6	3	0.56	0	-0.69	0	0.28	0	0.19	0
Shen Pons Rrv	Pons R		9.5	-20.5	-30.5	1.88	4	1.79	4	0.72	1	-1.46	0	-0.52	0	0.06	0
SII+ L	somatomotor operculum L	S2/dpIns	-46.5	-24.5	21.5	11.08***	9	10.11***	9	4.87**	8	-6.28*	0	-0.34	1	-1.17	0
SII+ R	somatomotor operculum R	S2/dpIns	45.5	-20.5	21.5												
SMA L	somatomotor paracentral lobule L		-12.5	-2.5	65.5	5.71**	9	5.83**	9	5.64***	9	-0.44	1	2.22	4	-0.42	0
SMA R	somatomotor paracentral lobule R		15.5	-2.5	65.5												
STH L	Midbrain L		-10.5	-10.5	-6.5	0.94	0	1.54	1	-0.53	0	-0.37	0	0.59	0	-0.66	0
STH R	Midbrain R		11.5	-10.5	-6.5												
striate ctx L	visual early L		-10.5	-84.5	1.5	3.02*	5	3.31*	5	1.51	3	-2.24	0	1.33	2	-0.58	0
striate ctx R	visual early R		13.5	-80.5	3.5												
STV L	parietal TPOJ L		-60.5	-50.5	17.5	4.84**	8	4.74**	8	1.97	4	-0.75	0	2.15	4	0.55	0
STV R	parietal TPOJ R		59.5	-42.5	19.5												
Subiculum L	Hippocampal Formation L		-20.5	-20.5	-18.5	3.76*	6	3.86*	6	1.73	2	-1.86	0	1.18	2	0.27	0
Subiculum R	Hippocampal Formation R		21.5	-20.5	-18.5												
superior IPL L	parietal inferior lobule L		-34.5	-58.5	39.5	4.2**	7	4.46**	7	1.71	2	-2.92	0	0.78	2	-1.26	0
superior IPL R	parietal inferior lobule R		39.5	-54.5	43.5												
Superior lateral BA8 L	cingulate dlPFC L		-16.5	25.5	53.5	2.73*	5	2.74*	5	0.68	3	0.02	2	1.41	3	-0.87	1
Superior lateral BA8 R	cingulate dlPFC R		17.5	27.5	55.5												
superior premotor L	somatomotor premotor L		-30.5	-6.5	57.5	5.58**	9	5.91**	9	2.99	6	-1.66	0	1.36	2	-0.94	0
superior premotor R	somatomotor premotor R		33.5	-4.5	55.5												
supplementary V3 L	visual dorsal L		-22.5	-86.5	21.5	2.66*	5	2.59*	5	1.12	1	0.17	1	1.78	3	0	0
supplementary V3 R	visual dorsal R		25.5	-82.5	25.5												

			MNI Coordinates			Hot-Rest (Target Body Site)		Hot-Warm (Target Body Site)		Hot-Warm x Target – Other Body Site		Imagine-Rest (Target Body Site)		Imagine-Warm (Target Body Site)		Imagine-Warm x Target – Other Body Site	
Parcel	Region	Pain Region	X	Y	Z	T	n	T	n	T	n	T	n	T	n	T	n
Tectum L	Midbrain L		-6.5	-34.5	-6.5	2.71*	5	3.22*	6	1.14	1	-2.16	0	0.44	0	-0.5	0
Tectum R	Midbrain R		5.5	-34.5	-6.5												
temporal pole L	temporal lateral L		-44.5	3.5	-32.5	3.63*	7	3.63*	7	2.39	3	-1.62	1	1.8	2	0.28	0
temporal pole R	temporal lateral R		41.5	7.5	-34.5												
Thal anterior L	Thal Anterior L		-4.5	-4.5	9.5	2.75*	3	2.7*	3	1.97	3	-1.7	0	1.1	2	0.33	0
Thal anterior R	Thal Anterior R		3.5	-2.5	9.5												
Thal dorsal L	Thal Lateral L		-8.5	-16.5	17.5	2.5*	3	2.63*	4	2.33	2	-0.73	0	1.6	1	1	0
Thal dorsal R	Thal Lateral R		7.5	-14.5	17.5												
Thal Intralaminar-anterodorsal L	Thal Intralaminar L	Thal IL	-0.5	-10.5	13.5	2.69*	5	2.45*	3	0.51	1	-0.58	0	0.69	2	-0.97	0
Thal Intralaminar-anterodorsal R	Thal Intralaminar R	Thal IL	3.5	-12.5	13.5												
Thal Intralaminar-antoventral L	Thal Intralaminar L	Thal IL	-2.5	-10.5	-0.5	2.52*	5	3.15*	6	2.64	5	-1.09	0	1.05	1	1.12	0
Thal Intralaminar-antoventral R	Thal Intralaminar R	Thal IL	3.5	-8.5	-0.5												
Thal Intralaminar-posterior L	Thal Intralaminar L	Thal IL	-10.5	-20.5	1.5	2.68*	4	2.86*	5	0.38	1	-1.89	0	0.29	1	-1.05	0
Thal Intralaminar-posterior R	Thal Intralaminar R	Thal IL	9.5	-18.5	1.5												
Thal Lateral-caudal L	Thal Ventral L	Thal VPLM	-18.5	-20.5	5.5	2.75*	5	2.73*	5	1.05	1	-1.67	0	1.23	1	-0.72	0
Thal Lateral-caudal R	Thal Ventral R	Thal VPLM	17.5	-18.5	5.5												
Thal Lateral-rostral L	Thal Ventral L		-10.5	-8.5	7.5	2.53*	4	2.42†	3	0.64	2	-3.54*	0	0.53	1	-2.44	0
Thal Lateral-rostral R	Thal Ventral R		11.5	-8.5	7.5												
Thal LGN L	Thal Posterior L		-24.5	-26.5	-2.5	3.07*	7	3.86**	7	2.84†	7	-1.89	0	0.25	0	0.82	0
Thal LGN R	Thal Posterior R		23.5	-26.5	-2.5												
Thal Medial L	Thal Medial L	Thal MD	-4.5	-14.5	5.5	3.92*	7	3.84*	7	1.49	1	-2.77†	0	0.94	2	-2.1	0
Thal Medial R	Thal Medial R	Thal MD	5.5	-14.5	5.5												
Thal MGN L	Thal Posterior L		-16.5	-28.5	-2.5	1.32	2	1.75	2	1.4	1	-1.46	0	0.47	1	0.41	0
Thal MGN R	Thal Posterior R		15.5	-26.5	-2.5												
Thal Pulvinar L	Thal Posterior L		-12.5	-28.5	9.5	3.71*	6	3.65*	6	2.9	5	-2.54	0	1.47	2	-0.6	0
Thal Pulvinar R	Thal Posterior R		13.5	-26.5	9.5												

Parcel	Region	Pain Region	MNI Coordinates			Hot-Rest (Target Body Site)		Hot-Warm (Target Body Site)		Hot-Warm x Target – Other Body Site		Imagine-Rest (Target Body Site)		Imagine-Warm (Target Body Site)		Imagine-Warm x Target – Other Body Site	
			X	Y	Z	T	n	T	n	T	n	T	n	T	n	T	n
TPOJ L	parietal TPOJ L		-50.5	-56.5	11.5	3.95*	5	3.81*	6	1.33	2	-1.36	0	2.07	3	-0.53	0
TPOJ R	parietal TPOJ R		51.5	-50.5	11.5												
V6 L	visual dorsal L		-16.5	-80.5	33.5	2.83*	5	2.69*	5	1.17	3	-1.01	1	1.46	2	-0.49	0
V6 R	visual dorsal R		19.5	-78.5	35.5												
V7 L	visual dorsal L		-24.5	-84.5	31.5	2.82*	5	2.8*	5	1.45	2	-0.09	0	1.54	3	0.22	0
V7 R	visual dorsal R		29.5	-82.5	33.5												
ventral intraparietal L	parietal superior lobule L		-34.5	-44.5	43.5	7***	9	7.36***	9	1.92	3	-3.46†	0	0.63	0	-1.2	0
ventral intraparietal R	parietal superior lobule R		35.5	-40.5	43.5												
vmPFC L	cingulate ACC mPFC L		-8.5	41.5	-2.5	3.11*	6	3.52*	6	0.6	2	-0.76	1	1.16	1	-0.39	0
vmPFC R	cingulate ACC mPFC R		7.5	41.5	-2.5												
vMT+ L	visual MT+ L		-46.5	-70.5	1.5	3.73*	5	3.4*	5	2.09	3	0.24	1	2.55	3	0.47	0
vMT+ R	visual MT+ R		47.5	-66.5	-0.5												
VMV L	visual ventral L		-24.5	-56.5	-6.5	3.36*	5	3.38*	5	1.7	4	-0.64	0	2.17	5	0.14	0
VMV R	visual ventral R		25.5	-54.5	-6.5												
VStriatum L	VStriatum L		-8.5	11.5	-6.5	3.03*	5	2.93*	5	1.49	2	-1.32	0	1.36	3	-0.02	0
VStriatum R	VStriatum R		7.5	11.5	-4.5												
VTA PBP SN L	Midbrain L		-8.5	-16.5	-12.5	2.07†	5	2.16†	5	1.22	0	-1.63	0	-0.35	0	-0.57	0
VTA PBP SN R	Midbrain R		9.5	-16.5	-12.5												
VVC L	visual ventral L		-30.5	-60.5	-14.5	3.31*	5	3.26*	5	1.25	3	1.18	2	2.96	4	-0.09	0
VVC R	visual ventral R		29.5	-54.5	-16.5												

Notes. Left (L) and Right (R) hemispheric regions are listed separately to define MNI XYZ coordinates separately, but were tested as a single region for pattern expression across body sites. Pain region column corresponds to voxel overlap within a-priori regions of interest in regions shown in Supplementary Table 3. P-values from all tests were FDR-corrected across 131 regional tests. n: Number of subjects with significant pattern expression post-FDR correction across 131 regions. aMCC MPFC: anterior midcingulate cortex/medial prefrontal cortex, aIns: anterior insula, mIns: medial insula, dpIns: dorsoposterior insula, S1: primary somatosensory cortex, S2: secondary somatosensory cortex, Thal: thalamus, VPLM: medial ventral posterolateral, IL: intralaminar, MD: mediodorsal, PAG: periaqueductal gray, RVM: rostral ventral medulla. †$p < .10$, *$p < 0.05$, **$p < 0.01$ and ***$p < .001$.

Supplementary Table S5: Representational similarity analysis by region

	Hot > Warm			Hot > Imagine			Imagine > Warm			(Hot, Imagine) > (Hot, Warm)			(Hot, Imagine) > (Imagine, Warm)			Target Body Site > Other Body Site		
ROI	T	Hot > Warm (n)	Hot < Warm (n)	T	Hot > Imagine (n)	Hot < Imagine (n)	T	Imagine > Warm (n)	Imagine < Warm (n)	T	(Hot,Imagine) > (Hot,Warm) (n)	(Hot,Imagine) < (Hot,Warm) (n)	T	(Hot,Imagine) > (Imagine,Warm) (n)	(Hot,Imagine) < (Imagine,Warm) (n)	T	Target > Other (n)	Target < Other (n)
55b	4.08**	9	0	-1.43	4	5	3.75*	9	0	2.90	6	3	3.61*	8	1	0.47	6	3
aCAU	2.68*	7	2	-0.58	5	4	2.85*	9	0	3.08	9	0	1.51	8	1	0.14	6	3
AMY BL CeM	3.58*	9	0	1.87	7	2	2.83*	8	1	0.70	4	5	0.57	4	5	0.80	5	4
AMY La	1.72	6	3	-0.83	5	4	2.59*	7	2	0.61	5	4	0.07	4	5	-1.58	3	6
anterior AAC	3.03*	9	0	0.24	6	3	3.31*	9	0	2.95	8	1	3.38*	9	0	-1.03	3	6
anterior agranular insula	4.19**	9	0	2.59	8	1	3.75*	9	0	1.86	7	2	2.65*	7	2	3.68*	8	1
anterior BA7	4.51**	9	0	2.69	7	2	4.51*	9	0	2.03	6	3	4.94*	8	1	3.80*	9	0
anterior IPL	6.51**	9	0	4.80*	9	0	3.46*	8	1	0.79	6	3	4.50*	8	1	0.81	6	3
anterior operculum	5.45**	9	0	3.39	9	0	2.98*	8	1	1.21	6	3	2.73*	7	2	0.79	6	3
anterior precuneus	3.55*	9	0	0.82	5	4	4.16*	8	1	1.27	4	5	2.29	6	3	-0.30	5	4
aPUT	2.83*	7	2	0.51	5	4	2.59*	8	1	1.67	6	3	1.41	6	3	0.68	4	5
auditory transition area	5.37**	9	0	3.49	8	1	3.31*	7	2	2.01	6	3	3.46*	9	0	3.96*	9	0
auditory+	3.70*	9	0	2.06	8	1	2.50*	8	1	1.18	5	4	1.62	6	3	7.13**	9	0
BA10	2.31*	8	1	0.75	6	3	2.98*	8	1	3.17	8	1	2.07	6	3	1.41	6	3
BA11	3.71*	9	0	1.39	6	3	4.22*	9	0	3.33	9	0	2.90*	8	1	0.23	5	4
BA13	3.26*	8	1	0.68	6	3	2.28*	6	3	1.24	6	3	0.56	5	4	0.64	6	3
BA44	3.67*	9	0	1.65	6	3	3.17*	9	0	2.05	8	1	2.45	7	2	-0.46	4	5
BA45	3.54*	9	0	1.05	6	3	3.37*	9	0	2.53	7	2	3.24*	7	2	0.97	5	4
BA46	3.96**	9	0	0.48	5	4	3.86*	9	0	3.15	8	1	2.95*	7	2	1.35	6	3
BA47	3.26*	9	0	0.67	5	4	2.84*	9	0	1.97	5	4	2.81*	6	3	1.55	7	2
BA5	5.41**	9	0	1.82	7	2	3.74*	9	0	0.71	4	5	1.52	7	2	3.09	8	1
BA9	2.60*	9	0	0.34	4	5	3.56*	9	0	2.77	8	1	2.73*	8	1	-0.62	4	5
Cblm CrusI CrusII VIIIb	3.49*	9	0	-0.84	5	4	3.17*	8	1	3.01	6	3	3.10*	8	1	1.36	6	3
Cblm I IV V VI	3.12*	9	0	0.92	6	3	3.67*	9	0	2.51	8	1	2.63*	9	0	2.60	7	2
Cblm vermis	2.26*	9	0	0.34	4	5	3.22*	8	1	2.30	7	2	2.12	7	2	0.07	4	5
Cblm VIIIa VIIb IX X	4.30**	9	0	0.89	6	3	3.42*	8	1	2.72	7	2	3.32*	8	1	1.93	6	3
Cholinergic nuclei	1.52	6	3	0.33	4	5	1.83	7	2	2.06	7	2	1.23	6	3	-0.19	3	6
cingulate motor area	4.24**	9	0	2.47	7	2	2.99*	8	1	1.26	4	5	1.80	7	2	2.91	7	2
Cranial nuclei	2.15*	7	2	0.13	5	4	2.11*	7	2	0.94	5	4	1.31	5	4	1.04	6	3
dmPFC	2.24*	9	0	0.21	5	4	3.96*	9	0	2.97	7	2	2.35	6	3	-1.07	4	5
dMT+	3.02*	8	1	0.18	5	4	3.71*	8	1	3.48	9	0	2.27	8	1	-0.25	5	4
dorsal intraparietal	3.53*	9	0	0.34	4	5	4.13*	9	0	2.59	7	2	4.43*	9	0	0.65	4	5
early auditory ctx a1	4.01**	9	0	2.26	7	2	2.43*	6	3	0.07	4	5	1.11	6	3	4.82*	9	0
extrastriate	2.60*	9	0	-3.33	0	9	3.89*	9	0	2.12	8	1	1.70	6	3	1.40	5	4
FFC	2.15*	7	2	-1.34	1	8	5.49*	9	0	1.84	6	3	1.99	6	3	1.74	6	3

ROI	Hot > Warm			Hot > Imagine			Imagine > Warm			(Hot, Imagine) > (Hot, Warm)			(Hot, Imagine) > (Imagine, Warm)			Target Body Site > Other Body Site		
	T	Hot > Warm (n)	Hot < Warm (n)	T	Hot > Imagine (n)	Hot < Imagine (n)	T	Imagine > Warm (n)	Imagine < Warm (n)	T	(Hot,Imagine) > (Hot,Warm) (n)	(Hot,Imagine) < (Hot,Warm) (n)	T	(Hot,Imagine) > (Imagine,Warm) (n)	(Hot,Imagine) < (Imagine,Warm) (n)	T	Target > Other (n)	Target < Other (n)
FOP1-BA43	8.85***	9	0	7.17**	9	0	3.19*	8	1	0.55	5	4	4.10*	8	1	3.56*	8	1
GPe	1.99*	6	3	-0.26	5	4	2.16*	8	1	2.13	6	3	2.02	7	2	2.37	7	2
GPi	1.89	5	4	2.11	7	2	1.55	5	4	1.32	5	4	1.38	6	3	1.72	7	2
Hipp	2.10*	9	0	1.47	7	2	3.41*	7	2	0.03	3	6	-0.12	5	4	0.27	5	4
hippocampal formation	2.74*	8	1	0.07	4	5	4.23*	8	1	2.59	7	2	1.61	6	3	0.74	4	5
hypothalamus	2.48*	8	1	1.57	6	3	2.58*	7	2	1.89	7	2	1.07	6	3	1.85	7	2
IFJ	2.86*	8	1	-1.03	5	4	3.31*	8	1	1.83	7	2	2.18	7	2	-0.80	4	5
IFS	4.64**	9	0	-0.04	5	4	3.16*	9	0	2.34	8	1	3.05*	8	1	-0.53	4	5
inferior dysgranular insula	5.88**	9	0	5.06*	8	1	3.37*	8	1	-0.46	3	6	1.36	7	2	3.39	8	1
inferior IPL	3.09*	9	0	0.66	6	3	3.43*	9	0	3.22	7	2	3.39*	8	1	-0.30	3	6
Inferior lateral BA8	3.13*	8	1	-0.88	3	6	3.32*	9	0	3.24	7	2	2.34	6	3	-0.37	4	5
inferior premotor	4.45**	9	0	2.13	7	2	3.70*	8	1	2.15	7	2	3.03*	8	1	0.42	6	3
inferior temporal sulcus	2.29*	9	0	0.36	5	4	4.50*	8	1	3.09	6	3	1.62	5	4	1.30	5	4
infralimbic	2.16*	8	1	0.83	6	3	2.22*	6	3	2.62	7	2	1.44	6	3	0.76	6	3
ISP1	3.66*	9	0	-0.31	4	5	3.98*	9	0	1.67	7	2	2.21	7	2	1.82	6	3
L PnO PnC	1.01	6	3	0.57	6	3	0.52	4	5	0.50	4	5	0.92	4	5	1.49	6	3
LC+	0.31	7	2	-0.70	3	6	0.86	6	3	0.85	4	5	0.56	4	5	-0.50	3	6
LO	3.08*	8	1	-2.44	2	7	5.39*	9	0	3.36	8	1	3.16*	9	0	2.83	8	1
M1	4.17**	9	0	0.87	7	2	3.02*	9	0	0.94	5	4	1.41	7	2	2.86	8	1
Medullary Raphe (Serotonergic)	0.19	1	8	-0.36	3	6	0.86	4	5	-0.84	4	5	-0.14	5	4	0.33	5	4
Medullary reticular formation	2.65*	9	0	1.62	7	2	1.34	4	5	1.88	6	3	2.58	6	3	0.53	3	6
midcingulate	4.25**	9	0	0.93	7	2	2.72*	9	0	1.95	6	3	2.50	8	1	1.93	8	1
middle IPL	3.44*	8	1	0.24	5	4	3.22*	9	0	1.92	6	3	2.84*	6	3	0.54	4	5
middle temporal gyrus	3.52*	9	0	0.18	4	5	3.26*	9	0	1.48	6	3	2.74*	7	2	0.70	5	4
MIP	3.43*	9	0	0.48	6	3	3.41*	9	0	1.55	6	3	2.80*	9	0	-0.56	4	5
OFC	2.30*	7	2	0.66	7	2	3.12*	8	1	1.59	7	2	0.87	5	4	0.30	4	5
Olivary complex	1.74	7	2	1.17	6	3	2.57*	8	1	1.33	6	3	1.24	5	4	-0.64	5	4
OP4	10.35***	9	0	6.99**	9	0	1.15	6	3	-1.59	3	6	3.31*	8	1	4.46*	8	1
PAG	1.47	7	2	0.29	6	3	1.69	7	2	-0.34	3	6	-0.07	4	5	-1.59	1	8
Parabrachial nuclei	2.42*	8	1	1.47	6	3	2.58*	7	2	1.29	6	3	1.12	6	3	-0.96	4	5
parietal occipital sulcus	3.43*	9	0	0.90	6	3	3.64*	9	0	2.13	8	1	2.31	7	2	0.75	5	4
pCAU	2.30*	8	1	-0.19	4	5	3.58*	8	1	2.11	8	1	1.89	8	1	-0.17	5	4
PCV	4.57**	9	0	-0.99	4	5	3.25*	9	0	2.62	7	2	3.71*	9	0	-0.51	4	5

	Hot > Warm			Hot > Imagine			Imagine > Warm			(Hot, Imagine) > (Hot, Warm)			(Hot, Imagine) > (Imagine, Warm)			Target Body Site > Other Body Site		
ROI	T	Hot > Warm (n)	Hot < Warm (n)	T	Hot > Imagine (n)	Hot < Imagine (n)	T	Imagine > Warm (n)	Imagine < Warm (n)	T	(Hot,Imagine) > (Hot,Warm) (n)	(Hot,Imagine) < (Hot,Warm) (n)	T	(Hot,Imagine) > (Imagine,Warm) (n)	(Hot,Imagine) < (Imagine,Warm) (n)	T	Target > Other (n)	Target < Other (n)
PeEc	3.59*	9	0	0.99	7	2	2.70*	9	0	1.20	5	4	0.49	5	4	2.87	7	2
PH RPH	2.52*	9	0	0.46	6	3	4.65*	9	0	2.12	7	2	2.63*	9	0	-0.25	2	7
PHA	2.55*	6	3	0.05	4	5	3.86*	9	0	2.46	6	3	2.47	8	1	0.57	5	4
PIR	5.70**	9	0	2.92	8	1	2.98*	8	1	-0.36	3	6	1.50	5	4	0.47	6	3
postcentral BA7	5.63**	9	0	1.79	7	2	4.99*	9	0	2.31	6	3	3.95*	8	1	2.13	7	2
posterior AAC	2.84*	9	0	0.45	5	4	3.13*	8	1	2.35	7	2	3.20*	9	0	-0.63	4	5
posterior BA7	3.05*	8	1	-0.44	4	5	3.17*	8	1	1.73	5	4	2.33	7	2	0.31	5	4
posterior granular insula	5.95**	9	0	5.14*	9	0	2.31*	7	2	-1.58	3	6	2.28	7	2	1.18	5	4
posterior IPL	3.41*	9	0	-1.15	3	6	4.06*	8	1	2.75	8	1	3.38*	7	2	0.33	5	4
posterior operculum	13.24***	9	0	10.58***	9	0	2.86*	6	3	-0.05	4	5	3.30*	7	2	4.49*	8	1
pPUT	1.97*	8	1	-1.15	4	5	2.42*	8	1	1.31	6	3	1.30	6	3	1.94	7	2
precuneus proper	3.09*	9	0	0.37	5	4	3.00*	8	1	1.96	5	4	2.06	7	2	-0.47	2	7
PSL	4.58**	9	0	3.42	7	2	3.63*	8	1	1.72	6	3	3.57*	8	1	-0.32	4	5
R PnO PnC	-0.11	4	5	0.55	4	5	-0.76	3	6	-0.17	3	6	0.37	3	6	-0.39	4	5
RN	1.83	6	3	-1.23	3	6	3.60*	8	1	1.41	6	3	1.90	7	2	-0.09	4	5
Rostral Raphe (Serotonergic)	0.98	6	3	-0.06	5	4	1.47	7	2	3.57	8	1	2.11	6	3	-1.14	2	7
Rostral reticular formation	2.34*	7	2	0.86	7	2	1.25	6	3	0.31	3	6	0.84	5	4	0.08	6	3
RSC	3.90**	9	0	0.69	6	3	2.77*	9	0	1.76	6	3	2.53	8	1	0.06	5	4
S1	4.33**	9	0	2.19	8	1	3.30*	9	0	1.27	6	3	2.04	7	2	3.71*	8	1
Shen Med	2.45*	9	0	1.59	6	3	1.91	6	3	1.74	6	3	2.55	6	3	1.10	6	3
Shen Midb Lc	2.44*	7	2	1.14	5	4	2.91*	9	0	1.98	8	1	0.84	5	4	-0.25	5	4
Shen Midb Lrd	2.94*	7	2	-0.12	5	4	4.87*	9	0	0.52	4	5	-0.62	2	7	0.54	5	4
Shen Midb Rcd	2.61*	9	0	0.83	6	3	4.10*	9	0	1.55	6	3	0.72	4	5	-1.93	3	6
Shen Midb Rrd	2.78*	8	1	0.51	6	3	3.13*	8	1	-0.19	3	6	-0.30	4	5	0.52	5	4
Shen Pons Lcd	2.83*	8	1	1.12	7	2	4.06*	9	0	0.90	6	3	0.80	4	5	0.13	3	6
Shen Pons Lrd	2.91*	9	0	1.92	7	2	2.61*	7	2	2.60	7	2	2.31	7	2	-1.32	4	5
Shen Pons Lv	1.78	7	2	0.76	6	3	1.05	5	4	-0.01	3	6	-1.01	5	4	-0.42	4	5
Shen Pons Rcd	2.46*	8	1	1.40	6	3	2.55*	8	1	1.36	6	3	0.89	6	3	0.70	5	4
Shen Pons Rcv	2.39*	6	3	1.50	7	2	1.17	5	4	-1.70	2	7	0.21	4	5	-0.14	4	5
Shen Pons Rrv	1.11	5	4	0.01	5	4	1.82	6	3	-0.09	3	6	-0.13	4	5	-1.07	3	6
SII+	7.25**	9	0	5.59**	9	0	1.92	6	3	-0.84	3	6	2.81*	7	2	4.69*	8	1
SMA	3.26*	9	0	-0.97	4	5	4.09*	9	0	2.52	8	1	2.71*	9	0	2.56	7	2
STH	1.71	6	3	0.61	6	3	1.43	6	3	1.58	6	3	0.82	7	2	0.06	5	4
strate ctx	2.85*	9	0	-2.26	4	5	4.39*	9	0	2.70	7	2	1.51	6	3	0.82	5	4
STV	5.52**	9	0	0.49	4	5	3.97*	8	1	2.27	7	2	3.48*	9	0	-0.58	6	3

	Hot > Warm			Hot > Imagine			Imagine > Warm			(Hot, Imagine) > (Hot, Warm)			(Hot, Imagine) > (Imagine, Warm)			Target Body Site > Other Body Site		
ROI	T	Hot > Warm (n)	Hot < Warm (n)	T	Hot > Imagine (n)	Hot < Imagine (n)	T	Imagine > Warm (n)	Imagine < Warm (n)	T	(Hot,Imagine) > (Hot,Warm) (n)	(Hot,Imagine) < (Hot,Warm) (n)	T	(Hot,Imagine) > (Imagine,Warm) (n)	(Hot,Imagine) < (Imagine,Warm) (n)	T	Target > Other (n)	Target < Other (n)
Subiculum	2.33*	9	0	1.16	7	2	3.70*	9	0	0.62	3	6	-0.48	3	6	-1.32	3	6
superior IPL	3.34*	9	0	0.23	6	3	2.31*	9	0	1.53	6	3	2.26	7	2	2.60	8	1
Superior lateral BA8	2.81*	9	0	-0.34	3	6	3.69*	9	0	3.15	8	1	2.87*	8	1	-0.72	3	6
superior premotor	4.37**	9	0	-0.27	3	6	3.51*	9	0	1.67	6	3	1.94	8	1	2.58	7	2
supplementary V3	2.80*	9	0	-2.99	2	7	3.83*	9	0	2.80	9	0	2.38	7	2	2.76	8	1
Tectum	3.02*	8	1	0.96	7	2	2.92*	8	1	1.20	5	4	1.26	5	4	1.38	5	4
temporal pole	3.15*	9	0	0.69	6	3	3.88*	9	0	2.81	6	3	2.79*	8	1	2.17	7	2
Thal anterior	1.92	7	2	1.14	7	2	2.62*	7	2	1.81	8	1	1.46	6	3	0.11	4	5
Thal dorsal	2.29*	8	1	1.09	7	2	3.91*	7	2	1.51	7	2	0.87	6	3	-1.70	2	7
Thal Intralaminar-anterodorsal	2.54*	7	2	1.84	7	2	2.81*	8	1	2.07	8	1	2.10	7	2	-0.65	4	5
Thal Intralaminar-antoventral	0.93	6	3	1.07	6	3	-0.06	3	6	-0.10	4	5	-0.40	4	5	1.92	7	2
Thal Intralaminar-posterior	2.68*	7	2	1.93	6	3	2.25*	7	2	-0.32	4	5	-0.15	4	5	2.84	7	2
Thal Lateral-caudal	2.52*	9	0	2.21	7	2	3.02*	8	1	1.70	6	3	1.85	8	1	0.34	6	3
Thal Lateral-rostral	2.20*	9	0	1.77	7	2	3.16*	7	2	1.43	8	1	1.63	5	4	0.01	4	5
Thal LGN	2.54*	7	2	1.38	7	2	2.87*	8	1	-1.86	2	7	-1.68	3	6	-1.62	3	6
Thal Medial	3.13*	8	1	2.00	7	2	3.73*	8	1	1.42	7	2	1.55	7	2	-2.50	2	7
Thal MGN	0.32	4	5	-1.45	4	5	1.97*	6	3	0.75	3	6	-0.25	2	7	0.22	5	4
Thal Pulvinar	2.54*	8	1	1.18	6	3	3.77*	9	0	1.22	6	3	1.01	4	5	-0.57	6	3
TPOJ	3.36*	9	0	0.79	5	4	4.44*	9	0	2.72	8	1	3.02*	9	0	0.59	5	4
V6	3.30*	9	0	-0.64	4	5	3.67*	9	0	2.27	7	2	1.27	6	3	0.95	7	2
V7	3.11*	8	1	-1.17	3	6	3.45*	9	0	2.95	7	2	2.87*	8	1	0.20	4	5
ventral intraparietal	4.35**	9	0	1.46	6	3	3.32*	7	2	1.25	7	2	3.38*	8	1	2.53	8	1
vmPFC	2.40*	9	0	0.62	4	5	3.07*	8	1	2.65	7	2	2.10	7	2	-0.66	4	5
vMT+	3.53*	9	0	-0.31	5	4	4.68*	9	0	3.78	9	0	3.47*	8	1	1.94	7	2
VMV	1.79	7	2	-2.35	3	6	2.86*	9	0	2.11	8	1	2.20	7	2	-0.35	5	4
VStriatum	2.95*	7	2	1.61	7	2	3.39*	9	0	2.14	6	3	1.15	5	4	-0.67	4	5
VTA PBP SN	3.98**	8	1	0.17	4	5	2.24*	7	2	0.91	5	4	0.36	5	4	0.20	5	4
VVC	2.86*	7	2	-2.56	1	8	4.67*	9	0	2.12	6	3	2.44	8	1	1.10	5	4

Notes. For each contrast, T is the signed one-sample t statistic (N = 9 participants) of the per-participant similarity difference (its sign indicates the direction of the effect). Asterisks denote significance in the named direction after false-discovery-rate (FDR) correction across the 131 parcels within that contrast (*q < .05, **q < .01, ***q < .001; one-tailed). The two count columns give the number of participants (of 9) whose similarity difference favored each direction. Rows are ordered alphabetically by region.
